# Selective small molecule PARG inhibitor causes replication fork stalling and cancer cell death

**DOI:** 10.1038/s41467-019-13508-4

**Published:** 2019-12-11

**Authors:** Jerry H. Houl, Zu Ye, Chris A. Brosey, Lakshitha P. F. Balapiti-Modarage, Sarita Namjoshi, Albino Bacolla, Daniel Laverty, Brian L. Walker, Yasin Pourfarjam, Leslie S. Warden, Naga Babu Chinnam, Davide Moiani, Roderick A. Stegeman, Mei-Kuang Chen, Mien-Chie Hung, Zachary D. Nagel, Tom Ellenberger, In-Kwon Kim, Darin E. Jones, Zamal Ahmed, John A. Tainer

**Affiliations:** 1 Departments of Cancer Biology and of Molecular and Cellular Oncology, University of Texas MD Anderson Cancer Center, 6767 Bertner Avenue, Houston, TX 77030 USA; 2 Department of Chemistry, The University of Arkansas at Little Rock, 2801S. University Ave, Little Rock, AR 72204 USA; 3 Department of Pharmaceutical Sciences, The University of Arkansas for Medical Sciences, 4301 West Markham Street, Little Rock, AR 72205 USA; 4 Harvard University, School of Public Health, Boston, MA 02115 USA; 5 Department of Chemistry, University of Cincinnati, 301 Clifton Ct, Cincinnati, OH 45221 USA; 6 The University of Texas MD Anderson Cancer Center, UTHealth Graduate School of Biomedical Sciences, Houston, TX USA; 7 Department of Biochemistry and Molecular Biophysics, Washington University School of Medicine, 660S. Euclid Avenue, Saint Louis, MO 63110 USA; 8 Graduate Institute of Biomedical Sciences and Center for Molecular Medicine, and Office of the President, China Medical University, Taichung, 404 Taiwan

**Keywords:** Biophysics, Cancer, Cell biology, Drug discovery, Molecular biology

## Abstract

Poly(ADP-ribose)ylation (PARylation) by PAR polymerase 1 (PARP1) and PARylation removal by poly(ADP-ribose) glycohydrolase (PARG) critically regulate DNA damage responses; yet, conflicting reports obscure PARG biology and its impact on cancer cell resistance to PARP1 inhibitors. Here, we found that PARG expression is upregulated in many cancers. We employed chemical library screening to identify and optimize methylxanthine derivatives as selective bioavailable PARG inhibitors. Multiple crystal structures reveal how substituent positions on the methylxanthine core dictate binding modes and inducible-complementarity with a PARG-specific tyrosine clasp and arginine switch, supporting inhibitor specificity and a competitive inhibition mechanism. Cell-based assays show selective PARG inhibition and PARP1 hyperPARylation. Moreover, our PARG inhibitor sensitizes cells to radiation-induced DNA damage, suppresses replication fork progression and impedes cancer cell survival. In PARP inhibitor-resistant A172 glioblastoma cells, our PARG inhibitor shows comparable killing to Nedaplatin, providing further proof-of-concept that selectively inhibiting PARG can impair cancer cell survival.

## Abstract (web summary)

PARG catalyzes the removal of poly(ADP-ribose) (PAR) from target proteins and executes critical functions in the DNA damage response. Here the authors provide structural and biological insight with small molecule PARG inhibitors and show that PARG inhibition sensitizes cells to ionizing radiation and kills cancer cells through replication fork defects.

## Introduction

Poly(ADP-ribose)ylation (PARylation) by PAR polymerase 1 (PARP1) and its reversal by PAR glycohydrolase (PARG) dictate multiple DNA damage responses^1–3^. PAR homeostasis regulates DNA damage responses by rapid and dynamic modulation of protein–protein and protein–DNA interactions essential for genomic integrity and cell survival^2^. Consequently, blocking PAR synthesis by PARP inhibitors (PARPi) impairs signaling and the repair of damaged DNA^4^ and causes centrosome amplification^5^. Accordingly, *PARP1*-null mice are more susceptible to carcinogenesis induced by DNA alkylating agents^6,7^. In contrast, PARP1 hyperactivation causes excessive PAR accumulation that is cytotoxic and triggers the release of apoptosis-inducing factor (AIF) from mitochondria, leading to cell death^8,9^. Similarly, mice lacking PARG show excessive PAR accumulation, resulting in early embryonic lethality and cell death^10^; whereas, PARG-deficient cells exhibit delayed repair of DNA single- and double-strand breaks (DSBs)^11,12^, plus aberrant cell cycle progression^13^.

PARG, a single gene alternatively spliced to generate five isoforms, is an endo- and exo-glycohydrolase that rapidly degrades PAR generated by PARP1 to coordinate DNA repair^14,15^. PARG hydrolyzes α(1′′-2′) O-glycosidic linkages in PAR chains to release ADP-ribose and oligo(ADP-ribose) chains that may signal genotoxic stress^1^. Bacterial, protozoan, and mammalian PARG structures^16–21^ reveal a macrodomain fold with conserved active site residues and ‘tyrosine clasp’ (Tyr clasp) unique to PARG enzymes, which suggest a common mechanism for PAR hydrolysis. However, PARG does not cleave the terminal ADP-ribosyl group linked to the target residue, which is turned over by macrodomain-containing mono(ADP-ribose) (MAR) hydrolases^22–24^. Yet, deletion of all PARG isoforms is embryonically lethal^10^, and deletion of the nuclear isoform causes sensitivity to alkylating agents and ionizing radiation (IR)^25^. PARG is recruited to DNA damage sites through interaction with PCNA and PARP1^26^, prevents IR-induced mitotic catastrophe^13^ and maintains replication fork stability in a BRCA2-dependent manner^27^. Conversely, PARG depletion can prevent replication fork restart^28^. *PARG* genetic knockdown sensitizes various cancer cells to chemotherapeutic agents and radiation^11,13,29,30^ and may cause tumor-specific killing in *BRCA2*-deficient cancer cells^27^. Furthermore, reduced PARG activity can decrease cytotoxicity associated with inflammation, ischemia, and stroke^3,31^ and reduces liver metastases of colon carcinoma^32^. PARG furthermore regulates proliferation and differentiation of dendritic cells and T cells via PARP/NF-κB in tumor metastases of colon carcinoma^4^. Yet, PARG inhibition as a therapeutic strategy is directly questioned by reports, showing that PARG downregulation enables PARPi resistance in BRCA2 and p53-null mouse cell-line^33^, and that PARG deficiency had little impact on BRCA1- and/or PTEN-deficient tumor cells^34^.

With these conflicting reports in mind, herein, we examined the TCGA database and found that in most human cancers PARG expression is upregulated, supporting the notion that PARG enzymatic function is required for tumorigenesis^35^. We sought to test PARG inhibition in cancer cells and found that although existing PARG inhibitors (PARGi) have proven valuable^29–31,36^, issues with potency, bioavailability, rapid clearance, selectivity, or defined mechanism of action can cloud their applications for biology. Reasoning that a bioavailable and specific PARG inhibitor would be an enabling tool, we employed chemical library screening that led to the discovery of thio-xanthine/methylxanthine derivatives as bioavailable, potent and specific PARG inhibitors. Our high-resolution crystal structures of human PARG bound to these methylxanthine inhibitors revealed detailed interactions that support a mechanism of competitive inhibition. In particular, we find that conformational switching of the Tyr clasp that distinguishes PARG from MAR and other glycohydrolases enables specific engagement of methylxanthine derivatives in the PARG active site. Crucially, our cell-based assays show effective cellular activity, including PAR accumulation in cells, inhibited PARP1 dePARylation and increased γH2AX foci formation. Indeed, H2AX phosphorylation levels in cells treated with PARGi uncover synergistic sensitization to IR. Furthermore, PARGi induces replication fork defects resembling those reported with PARG knockdown^36^, and causes cellular sensitivity in PARP1-inhibitor-resistant cells. Moreover, our compounds are PARG-selective and do not target other cellular glycohydrolases at the relevant doses, in contrast to other reported PARGi’s. Our selective inhibitor reveals PARG functions that support and extend previous genetic findings, providing insights into PAR metabolism following IR damage and establishing these PARGi as enabling tools for investigating PAR biology with potential for advanced therapeutic strategies.

## Results

### Identification of specific PARG inhibitors

**Fig. 1:**
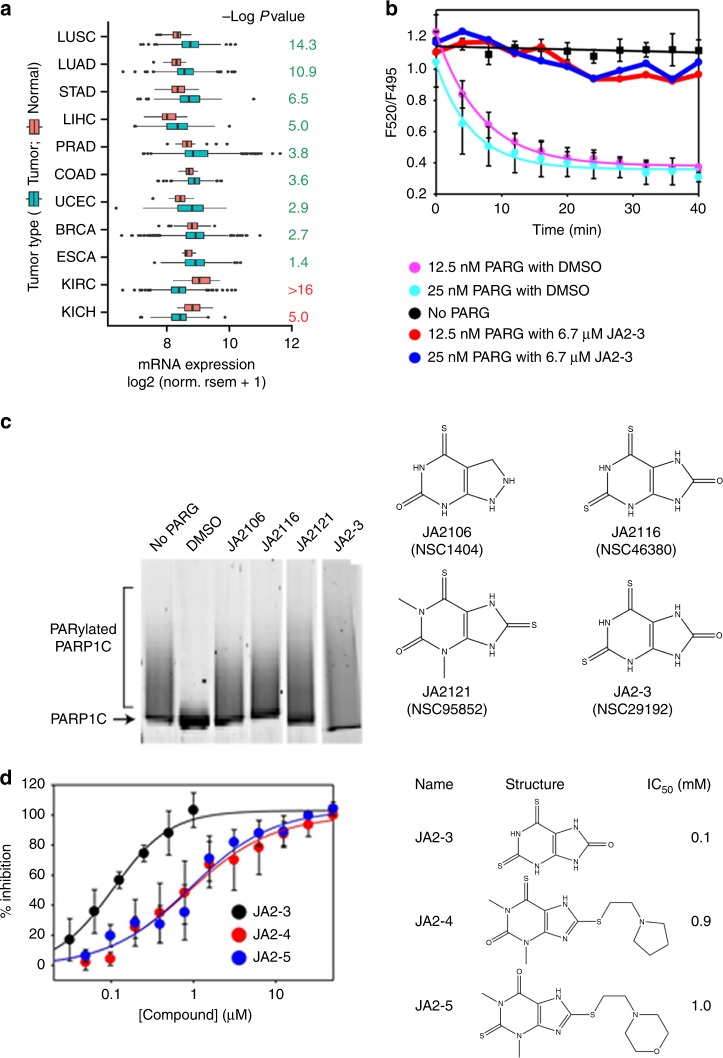
Identification of thio-xanthine/methylxanthine derivatives as PARG inhibitors. **a** PARG expression is upregulated in the majority of human cancers. Analysis of PARG expression in TCGA tumors and matched normal tissues. Log *p*-values were from Wilcoxon tests with greater values indicating stronger differences; PARG expression was higher in tumors than in matched controls in all tumor types (green) except in kidney malignancies (KIRC and KICH, red). Datasets with >10 normal samples and significant p-values were included. Box plots display the interquartile range (IQR) from Q1 to Q3 (25–75% percentiles), median (centre line), whiskers extending to the minimum (Q1–1.5*IQR) and maximum (Q3 + 1.5*IQR) and outliers (dots). **b** the raw data of HTS for JA2-3. The high reproducibility of the TR-FRET assay for PARG activity is reflected by a Z-factor of 0.6–0.8 (Supplementary Fig. 2), this kinetic high-throughput screen was performed against the NCI Diversity Set II library (1990 compounds in seven 384-well plates) at 6.7 μM compound concentrations and two PARG concentrations (12.5 nM and 25 nM). **c** Thio-xanthine/methylxanthine derivatives are potent PARG inhibitors. De-PARylation of PARP1C by PARG (25 nM) in the presence of the designated compound at 6.7 μM (left panel); the structure of the chemicals (right panel), among five chemotypes identified from the HTS (Supplementary Fig. 3). The JA2 xanthine/methylxanthine series was selected as the lead pharmacophore, based on its structural similarities to adenine, potency in vitro, and favorable drug-like characteristics. Dose-dependent inhibition of PARG activity by the JA2 series compounds was quantitatively analyzed using a gel-based PARG activity assay (Supplementary Fig. 4). **d** Three representative HTS hits of the JA2 chemotype (JA2-3, JA2-4, and JA2-5) show a potent PARG inhibition with the sub-micromolar range of IC_50_ values. Source Data are provided as a Source Data file.

Our TCGA database analysis revealed that in most human cancers PARG expression is upregulated (Fig. 1a). Importantly, PARG enzymatic function can be required for tumorigenesis^35^. Therefore, we sought to identify a selective PARG inhibitor through our robust PARG enzyme kinetic assay^37^, which quantitatively monitors real-time PAR-mediated PARP1-XRCC1 interactions and disassembly in a multi-well format. A strong TR-FRET signal was generated upon assembly of the Tb^3+^-chelated XRCC1 (FRET donor) with PARylated PARP1, which incorporates fluorescein-labeled ADP-ribose moieties into PAR chains (FRET acceptor). The addition of PARG caused a time-dependent loss of the TR-FRET signal by reversing PARylation and disassembling the PARP1-XRCC1 complex (Supplementary Fig. 1)^37^.

We screened the National Cancer Institute (NCI) Diversity Set II library (1990 compounds) against rat PARG. Then, human PARG was used to validate and optimize PARG inhibitors. The accuracy of the screen was determined by the calculated Z-scores of control reactions, which were between 0.6 and 0.8 throughout the plates, indicative of high reproducibility (Supplementary Fig. 2). Two different enzyme concentrations (12.5 nM and 25 nM) were used in subsequent analyses, with the rationale that potent inhibitors would inhibit PARG activity at both enzyme concentrations, while weaker inhibitors would inhibit PARG activity only at the lowest PARG concentration. The rate of TR-FRET signal loss in the presence of each candidate inhibitor was compared to that of control reactions, containing DMSO only (Fig. 1b). Initially, we selected 104 compounds that inhibited PARG enzymatic activity at both enzyme concentrations. Thirty-nine of these compounds were discarded as they altered fluorescence intensity of donor or acceptor (F_520_ and F_495_) by >40% at the first time point. An additional twelve compounds containing metals (e.g., Pt, Hg, or As) and five compounds resembling short chain fatty acids and steroids were also discarded, leaving 51 candidate inhibitors (hit rate of 2.6%), including 12 potent hits showing ~100% inhibition at both PARG concentrations (Supplementary Table 1). For the selected compounds, gel-based assays were performed to directly monitor inhibition of PARP1 dePARylation by PARG (Fig. 1c).

Most initial hits from the High Throughput Screening (HTS) contained 5- or 6-member rings and were grouped into five main chemotypes (Supplementary Fig. 3): succinimide (JA1: 2 hits), xanthine/methylxanthine (JA2: 13 hits), hydroquinone (JA3: 13 hits), nitrobenzofuranzan (JA4: 9 hits), and benzyl-sulfonamide (JA5: 14 hits). A total of 34 compounds containing representative hits and available analogues from each chemotype were then analyzed in dose-response curves, using both the TR-FRET assay and the gel-based assay. We confirmed that 19 compounds from five chemotypes inhibit PARG activity at concentrations of 0.1–10 μM (Supplementary Fig. 4 and Supplementary Table 2).

The TR-FRET assay is an indirect measurement of PARG activity, as it monitors PARG-mediated reversal of a PAR-dependent PARP1-XRCC1 interaction. Furthermore, loss of the TR-FRET signal was not detected until all PAR chains were degraded to lengths shorter than 7 ADP-ribose units^37^. Nonetheless, this assay enabled the measurement of steady-state PAR turnover rate, which was adequate for detecting enzyme inhibitors. In contrast, the gel-based assay directly monitors enzymatic conversion of PARylated PARP1 to an unmodified product by PARG, enabling a semi-quantitative measurement of enzyme inhibition (Supplementary Fig. 4). The IC_50_ value of a known PARG inhibitor, adenosine diphosphate (hydroxymethyl)pyrrolidinediol (ADP-HPD), determined by the gel-based assay (1.07 μM, Supplementary Fig. 5), which was in agreement with its published values (0.1–1.4 μM)^20,38,39^. Therefore, we chose the gel-based PARG activity assay in subsequent studies.

Inspection of the five identified chemotypes of PARG inhibitors (Supplementary Fig. 3) suggests that the JA2 series of xanthine/methylxanthine derivatives were the most attractive for several reasons. First, these compounds are structurally analogous to the adenine base of ADP-ribose (ADPR), an enzymatic product of PARG and have good potential for site-specific binding. Second, xanthine/methylxanthine-based compounds typically show good bioavailability, as evidenced by caffeine and the widely used bronchodilator, theophylline^40^. Third, the fused 5- and 6-membered rings of the xanthine core are synthetically tractable for analoging^40^. Most importantly, three thio-xanthine/methylxanthine derivatives (JA2-3/NSC29192, JA2-4/NSC99657, and JA2-5/NSC99667) were among the most potent dose-dependent inhibitors of PARG activity as assessed by HTS, with IC_50_ values between 0.1 and 1.0 μM (Fig. 1d).

### JA2-4 specifically binds to the hPARG adenine-binding pocket

To identify the binding sites of JA2-3, JA2-4, and JA2-5, these compounds were soaked into crystals of human PARG (hPARG) catalytic domain (residues 448–976). Crystals of hPARG diffracted to high resolution, and the crystal packing arrangement was more amenable to binding compounds in the enzyme active site^20^, compared to crystals of rat and mouse PARG^18,19^. The hPARG active site has two deep pockets that specifically engage the adenine and diphosphate group of ADPR. Tyr795 at the tip of the Tyr clasp points towards the heart of the active site and simultaneously interacts with the adenine and diphosphate moieties.

**Fig. 2:**
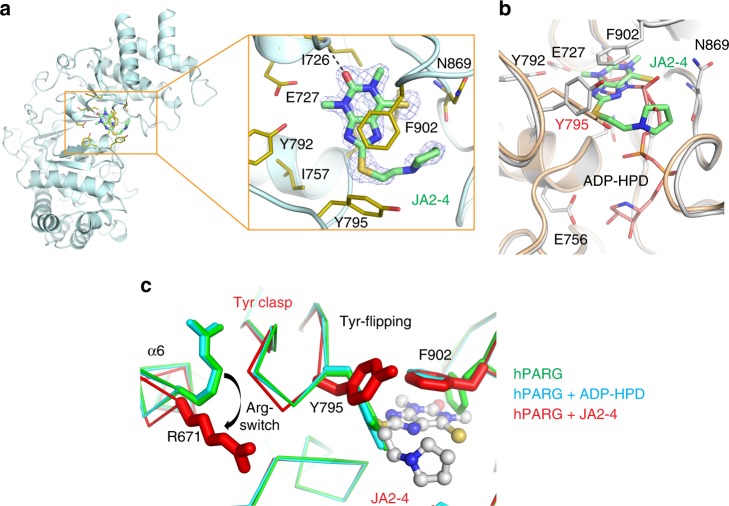
JA2-4 binds to the adenine-binding pocket of hPARG by virtue of the flexibility of the Tyr clasp. **a** 1.7 Å crystal structure of human PARG bound to JA2-4 reveals specific and extensive inhibitor interactions with the adenine-binding pocket. The 6´-thiocarbonyl sulfur of JA2-4 was assigned by comparing the size and orientation of the electron density to that of the corresponding 6´-carbonyl oxygen of JA2120 (Fig. 3b). JA2-4 is shown with a Fo-Fc map (contoured at 2σ) that was calculated prior to the addition of JA2-4 to the model. JA2-4 bound PARG catalytic domain shown in the enlarged box. **b** A structural comparison of the binding interactions of JA2-4 and ADP-HPD is consistent with a competitive inhibition mechanism for JA2-4. The structure of hPARG bound to ADP-HPD (wheat) is overlaid onto that bound to JA2-4 (white). The methylxanthine core of JA2-4 makes extensive interactions and occupies the same binding site as the adenosine moiety of ADP-HPD and. **c** The Tyr clasp changes conformation to enable binding of JA2-4. JA2-4 binding shifts the position of the Tyr clasp in concert with the rotation of the Tyr795 side chain and rearrangement of Arg671. The structural flexibility of the Tyr clasp creates room for JA2-4 and facilitates interactions with Asn869 and I726 that contribute to potent inhibition of PARG activity (IC_50_ of 0.9 μM).PDB code: 6O9X (PARG/JA2-4), 6O9Y (PARG/JA2-8), 6OA0 (PARG/JA2-9).

**Fig. 3:**
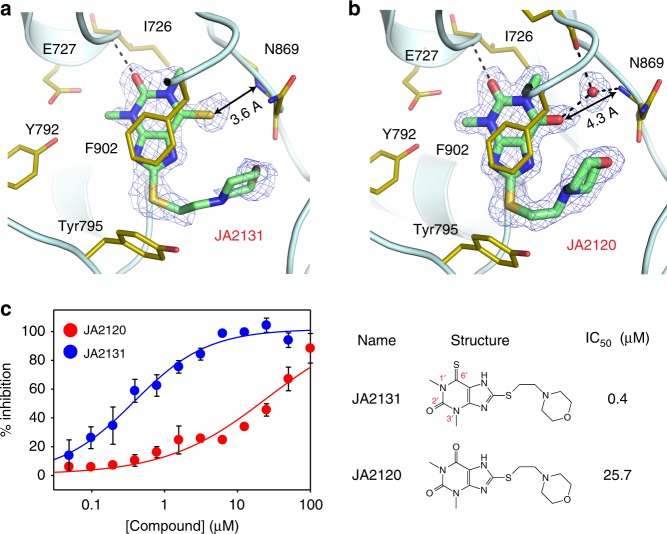
The 6´-thiocarbonyl group contributes strongly to the potency. **a, b** Crystal structures of human PARG bound to JA2131 **a** and JA2120 **b**. JA2131 and JA2120 occupy the adenine-binding pocket of hPARG in the same orientation as JA2-4. The 6´-thiocarbonyl of JA2131 makes a direct van der Waals contact with the main chain amide of Asn869 (3.6 Å) **a**, whereas the 6´-carbonyl of the less potent inhibitor JA2120 makes water-mediated hydrogen bonds to the main chain amide of Asn869 (4.3 Å) and the main chain carbonyl of Phe902 **b**. These findings indicate that the 6´-thiocarbonyl group of JA2-4 series inhibitors increases potency by introducing a direct interaction with hPARG. **c** The structure-activity relationships (SAR) for JA2-4 series inhibitors. The direct interaction between the 6´-thiocarbonyl group of JA2131 and hPARG increases potency more than 50-fold in comparison to JA2120. PDB code: 6OA1 (PARG/JA2120), 6OA3 (PARG/JA2131). Source Data are provided as a Source Data file.

The crystal structure of JA2-4 bound to hPARG refined at 1.7 Å resolution, (Fig. 2 and Supplementary Tables 3) identified a single JA2-4 molecule in the adenine-binding pocket. The 6´-thiocarbonyl sulfur of JA2-4 was assigned by the size and orientation of the electron density compared to that of the corresponding 6´-carbonyl oxygen of JA2120 (Figs. 2a and 3b). JA2-4 makes extensive site-specific interactions with hPARG, consistent with its potent inhibitory activity (IC_50_ of 0.9 μM) (Fig. 1d). The methylxanthine head of JA2-4 makes π-stacking interactions with Phe902 side chain. The tail of JA2-4 extends from the 8´-position of the xanthine ring and is highly curved, which serves to make van der Waals contacts with Asn869. The terminal pyrrole of JA2-4 is rotated ~90° and is perpendicular to the xanthine ring system. Importantly, most protein-JA2-4 interactions come from the conserved macrodomain fold. Despite the large conformational change (described below), the Tyr clasp generates limited direct contact with JA2-4; only the C_β_ of Tyr795 interacts with the thioether group of JA2-4 (Fig. 2a). JA2-4 has a surface area of 484 Å^2^ of which 89% (432 Å^2^) is masked by direct contacts with hPARG, which can explain the relatively high potency of the JA2-4 series compounds.

Structural superposition of hPARG bound to JA2-4 and ADP-HPD revealed that the methylxanthine core of JA2-4 occupies the same binding site as the adenine of ADP-HPD (Fig. 2b), consistent with a competitive block of substrate binding activity. However, the ring system of the xanthine core is flipped horizontally and rotated ~15° clockwise, relative to the adenine ring of ADP-HPD, and translated 0.8 Å towards the tip of the Tyr clasp. This distinctive ring orientation reflects a direct interaction of the 6´-thiocarbonyl group with the main chain amide of Asn869 (Fig. 2a). As predicted, a thiol substituent at the 6´ position of the methylxanthine pharmacophore greatly increases potency (Fig. 3). Additionally, the 2´-carbonyl oxygen of JA2-4 hydrogen bonded with the main chain amide of Ile726. This interaction is not made by ADP-HPD, and it displaces the methylxanthine ring by 0.8 Å with respect to the adenine ring of ADP-HPD (Fig. 2a, b). The 2´-carbonyl oxygen of JA2-4 also serves as a pivot point for rotation of the methylxanthine ring. Collectively, these structures support specific and potent inhibition of PARG activity by JA2-4 and suggest a mechanism of competitive inhibition by JA2 series xanthine/methylxanthine derivatives.

### Identification of key contact points of JA2-4

The most striking feature of the JA2-4 interaction with hPARG is a significant change in the Tyr clasp conformation (Fig. 2c). The JA2-4 binding site is obstructed by the side chain of Tyr795 in the unbound hPARG and in hPARG bound to ADP-HPD. To accommodate JA2-4, the Tyr795 side chain is flipped out from the adenine-binding pocket (with ~90° rotation), which positions the thioether of JA2-4 in contact with C_β_ of Tyr795. Furthermore, the interaction of JA2-4 with Tyr795 pushes on the tip of the Tyr clasp, which is displaced ~0.9 Å towards the N-terminal helical bundle (Fig. 2c).

Twisting of the Tyr clasp induced by JA2-4 binding involves several new interactions with a neighboring helix α6 (Fig. 2c). Most notably, JA2-4 binding caused the Arg671 side chain to swing ~9 Å towards the tip of the Tyr clasp, where it makes an electrostatic interaction with Glu797 side chain and hydrogen bonds to the main chain carbonyl of Ala750 (not shown in this view). This Arg671 pivoting motion constitutes a switch that serves as a backstop to secure the Tyr clasp in contact with the N-terminal helical bundle, which is ~30 Å away from Tyr795. Taken together, this previously undetected structural switching of the Tyr clasp may explain the basis of JA2-4 binding and its inhibition of PARG activity.

### Structure-based design improved PARGi potency and selectivity

We examined the structure-activity relationships (SAR) of JA2-4 analogs (Fig. 3) in competitive inhibition experiments. JA2131/NSC98003 contains a terminal morpholine in place of the pyrrole of JA2-4 and shows improved activity, which may derive from a polar interaction mediated by the 1´-oxygen of the morpholine (Fig. 3a, c). The 6´-thiocarbonyl group of JA2131 is another driver of inhibitory potency. Substitution with a carbonyl at this position decreases potency more than 50-fold (IC_50_ of JA2131 = 0.4 μM, IC_50_ of JA2120/NSC81474 = 25.7 μM, Fig. 3c), even though the 6´-carbonyl oxygen of the methylxanthine of JA2120 can make a hydrogen bond with the main chain amide of Asn869. The position of the thiocarbonyl group can be changed without the loss of activity because JA2131 (6´-thiocarbonyl; IC_50_ of 0.4 μM) (Fig. 2c) and JA2-5 (2´-thiocarbonyl; IC_50_ of 1.0 μM) (Fig. 1d) exhibit comparable potencies.

To further test the molecular basis for these SAR data, crystal structures of hPARG bound to JA2131 and JA2120 were determined at 1.9 and 1.8 Å resolution, respectively (Fig. 3a, b). JA2131 and JA2120 bind to the adenine-binding pocket of hPARG in the same orientation as JA2-4. As for JA2-4, the 6´-thiocarbonyl of JA2131 makes a direct van der Waals contact with the main chain amide of Asn869 (3.6 Å) from the conserved macrodomain fold (Fig. 3a). The morpholine oxygen of JA2131 appears to accept a hydrogen from the side chain amine of Asn869, explaining the modest increase in potency compared to JA2-4 (Fig. 2a).

In contrast, the distance between 6´-carbonyl of JA2120 and the main chain amide of Asn869 is significantly longer than that of 6´-thiocarbonyl of JA2131 and is beyond the range of direct polar or van der Waals interactions (4.3 Å) (Fig. 3b). Instead, a bound water molecule, which is absent in the JA2131- and JA2-4-bound structures, is positioned between JA2120 and Asn869. The bound water molecule relays an indirect hydrogen bond from 6´-carbonyl of JA2120 to the main chain amide of Asn869 and to the main chain carbonyl of Phe902 (Fig. 3b). A bound water molecule is also present at this site in structures of hPARG and hPARG bound to ADP-HPD. Notably, the 6´-thiocarbonyl group of JA2-4 series inhibitors displaces this water molecule to form improved interactions with the adenine-binding pocket of hPARG.

### PARG regulates PARP1 modification in cells

**Fig. 4:**
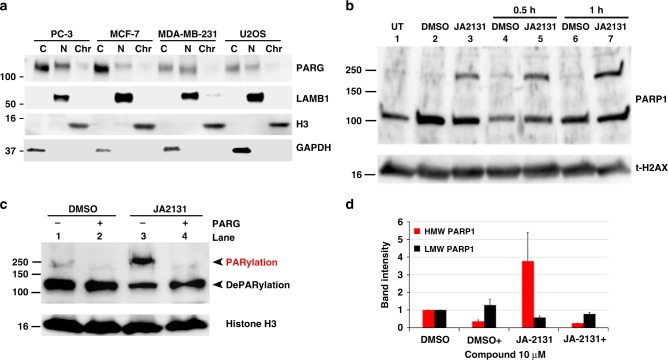
JA2131 induces hyperPARylation of PARP1**. a** Subcellular PARG protein expression patterns in cultured cells. Subcellular fractionated lysates were immunoblotted with anti-PARG, (upper panel) followed by nuclear (N) marker anti- Laminin Subunit Beta 1 (LAMB1, upper middle panel), chromatin (Chr) marker anti-Histone H3 (H3, lower middle panel) and the cytoplasmic (C) marker anti- Glyceraldehyde 3-phosphate dehydrogenase (GAPDH, lower panel) **b** PC3 cells were treated with DMSO or PARG inhibitor JA2131 (2131) for 2 h then irradiated with 7 Gy and allowed to recover for 0.5 h or 1.0 h before lysis and subcellular fractionation. Chromatin bound cell-extracts were analyzed with anti-PARP1 antibody (upper panel) followed by probing with total Histone H2AX (t-H2AX, lower panel) as the loading control. **c** DMSO or 2131-treated PC3 cells were irradiated and recovered for 2 h as above, chromatin fractions were then incubated with or without purified PARG enzyme (±PARG) for 30 min at 37 °C then immunoblotted with anti-PARP1 (upper panel) and then with anti-histone H3 (lower panel) as loading control. **d** Quantitative analysis of western blot of **b**, where expression of PARP1 levels in DMSO treated was normalized to 1. High molecular weight (HMW) in red PARP1 is significantly decreased in the presence of purified truncated PARG protein. The low molecular weight in black PARP1 bands are not significantly affected, *n* = 3. Plus denotes added purified PARG enzyme. Source Data are provided as a Source Data file.

To identify suitable cell lines to test PARGi, we evaluated PARG protein expression patterns on a panel of prototypic cancer cell lines. Cells were fractionated into cytoplasmic, nuclear and chromatin, and then PARG expression and localization were evaluated using an anti-PARG antibody (Fig. 4a). A varying level of PARG was detected in three cellular compartments. In prostate adenocarcinoma (PC3) and breast mammary epithelial (MCF-7) cells, PARG level was the highest in the cytoplasm, followed by the nucleus, with the lowest seen in the chromatin fraction. The triple-negative MDA-MB-231 breast cancer and U2OS osteosarcoma cells showed comparable expression between cytoplasm and nucleus. The PC3 cell line, with one of the highest levels of PARG expression, was selected for our initial PARGi testing. PC3 cells were treated with either DMSO or JA2131 for 2 h, then exposed to 7 Gy of IR and allowed to recover for a designated time period before harvesting and cell fractionation. Chromatin fractions of the cell lysates were analyzed by immunoblotting with PARP1 specific antibody (Fig. 4b), and the PARylation states of PARP1 were monitored as a marker for PARG inhibition. A high molecular weight (HMW) PARP1-containing band in the chromatin fractions, suggestive of hyper-PARylated PARP1, was only seen in cells treated with JA2131 PARGi. Cells treated with both JA2131 and IR displayed enhanced levels of PARylation-trapped PARP1 (Fig. 4b, 0.5 h versus 1.0 h). In these cells, PARylated PARP1 in response to PARG inhibition appeared as a discrete HMW (DHMW) proteins with limited smearing, as observed previously^41^. The DHMW was only seen when chromatin fractions were immunoblotted with the PARP1 antibody; by contrast, nuclear fractions and total cell lysates showed a contiguous smear, typically associated with PARylated PARP1. To assess whether DHMW was due to PARylation and not any other post-translational modifications, chromatin fractions of the PARGi-treated PC3 cell-lysates were treated with purified PARG, followed by anti-PARP1 western blotting. This led to the restoration of LMW PARP1, indicating PARylation was indeed causing the DHMW (Fig. 4c, d). Thus, a dynamic equilibrium between DHMW and LMW nuclear PARP1 exists in cells, and PARGi shifts this equilibrium towards DHMW. We conclude that PARGi induces PARP1 DHMW as a result of persistent PARylation, which can be reversed in vitro with exogenous purified PARG, suggesting a selective role of PARG in the dynamic regulation of PARP1 modification and signaling.

### PARGi induces PAR accumulation and γH2AX foci in nuclei

**Fig. 5:**
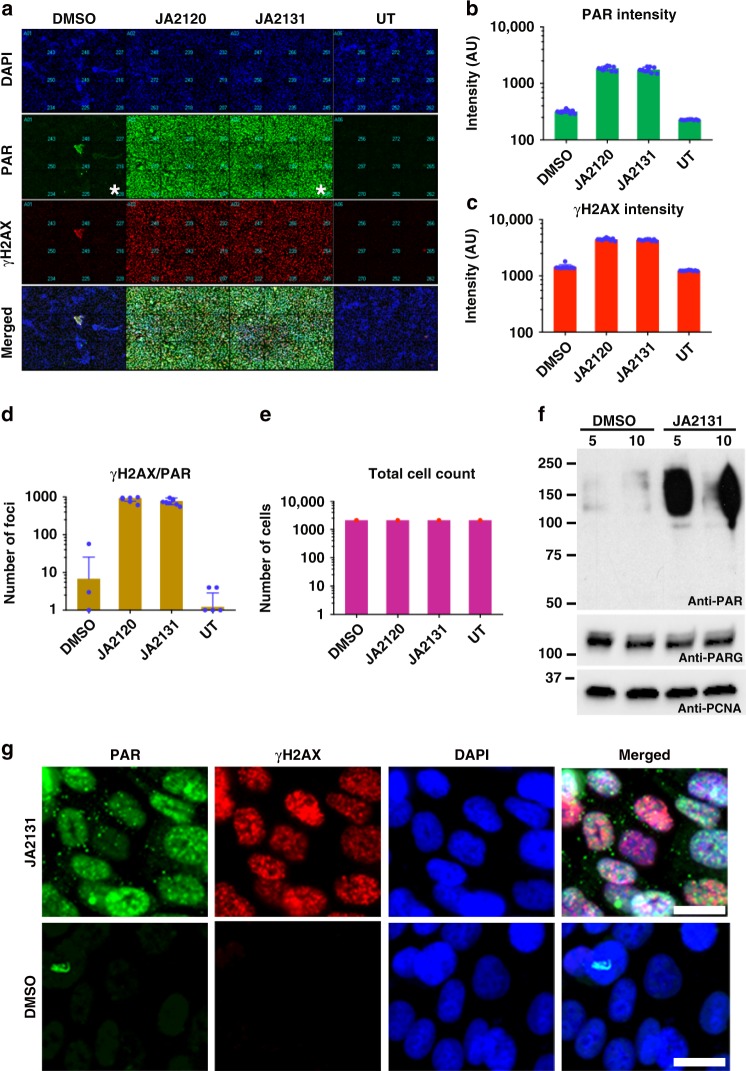
PARGi sensitizes cells to IR damage. **a** High level of PAR accumulation and γH2AX foci formation in cells exposed to PARGi. PC3 cells treated with DMSO or PARG inhibitors (JA2120 or JA2131) for 2 h, irradiated with 7 Gy, recovered for 1 h were fixed and immuno-stained with Poly(ADP)-Ribose (PAR, green) and γH2AX (red) antibodies, nucleus stained with Hoechst (blue). Cells were analyzed with quantitative high-content imaging (**b**–**e**). Quantitative analysis of PAR intensity (**b**), γH2AX intensities (**c**), the number of cells showing PAR / γH2AX co-localizations (**d**), and nucleus count for the total number of cells analyzed for each group (**e**). **f** Immunoblotting of PARGi JA2131-treated PC3 cells showing inhibitor-induced cellular PARylation. Cells were treated with JA2131 for 2 h followed by 7 Gy IR, then allowed to recover for 1 h before lysis. Total cell lysates were immunoblotted with anti-PAR (upper panel) followed by anti-PARG (middle panel) and Anti-PCNA (lower panel) as loading controls. **g** Enlarged, individual, representative images taken from one quadrant of the 3 × (3 × 3) square shown in **a** and the region marked with an asterisk. This represents the quality of the image used to perform quantification for foci and colocalization calculations. Anti-PAR (green), Anti-γH2AX (red) and Hoechst 33342 (blue). Scale bar 25 μm. Note that the image contrast was quantitatively controlled and equal for both sets of data, see Supplementary Fig. 6 for independently contrast-adjusted images. Source Data are provided as a Source Data file.

As we found that inhibition of PARG increases PARP1 in a PARylated DHMW form, we tested whether our PARGi augments the level of PAR accumulation and of double-stranded DNA (dsDNA) breaks in cells treated with IR. Following IR, cells treated with JA2131 showed a significant increase in PAR accumulation in comparison to untreated cells (20-fold increase with JA2131). DMSO treated cells showed a modest post-IR PAR accumulation that was comparable to the untreated cells (Fig. 5a, b, DMSO versus JA2131). A low magnification 3 × 3 image montage showed distinct PAR accumulation and γH2AX foci formation (Fig. 5a and Supplementary Fig. 6). Quantification revealed that PAR accumulation coincides with γH2AX staining and foci formation, which was >10-fold higher in PARGi-treated cells than in control cells (Fig. 5c). Furthermore, at least 50% of cells showed colocalization of PAR and γH2AX foci (Fig. 5d, γH2AX/PAR foci against total cell counts Fig. 5e). Western blot analyses also indicated that JA2131-induced PAR accumulation (Fig. 5f). Inspection of images at high magnification validates the automated quantification of observed JA2131-induced PAR/γH2AX colocalization. DMSO treated cells display diffuse, low-level PAR staining in the nucleus of cells with limited γH2AX foci. However, for cells treated with the PARGi JA2131, PAR staining was localized to distinct nuclear foci that also contained the phosphorylated H2AX (γH2AX) (Fig. 5g). We saw some γH2AX foci in DMSO treated cells, but only if the contrast was independently adjusted (see Supplementary Fig. 6a, b, bottom panels). Indeed, PAR accumulation caused by the treatment of cells with IR was further augmented by the PARGi, indicating that it functions as a DNA damage sensitizer. It was previously reported that genetic knockdown of *PARG* results in sensitization of cancer cells to chemotherapeutic agents and radiation^11,13,29,30^, and tumor-specific killing in *BRCA2*-deficient cancer cells^27^. Here, we reveal an effective PAR accumulation that coincides with elevated γH2AX following treatment with a novel PARGi in PC3 cells that have intact *BRCA1* and *BRCA2* genes^42^.

### JA2131 kills cancer cells through selective PARG inhibition

**Fig. 6:**
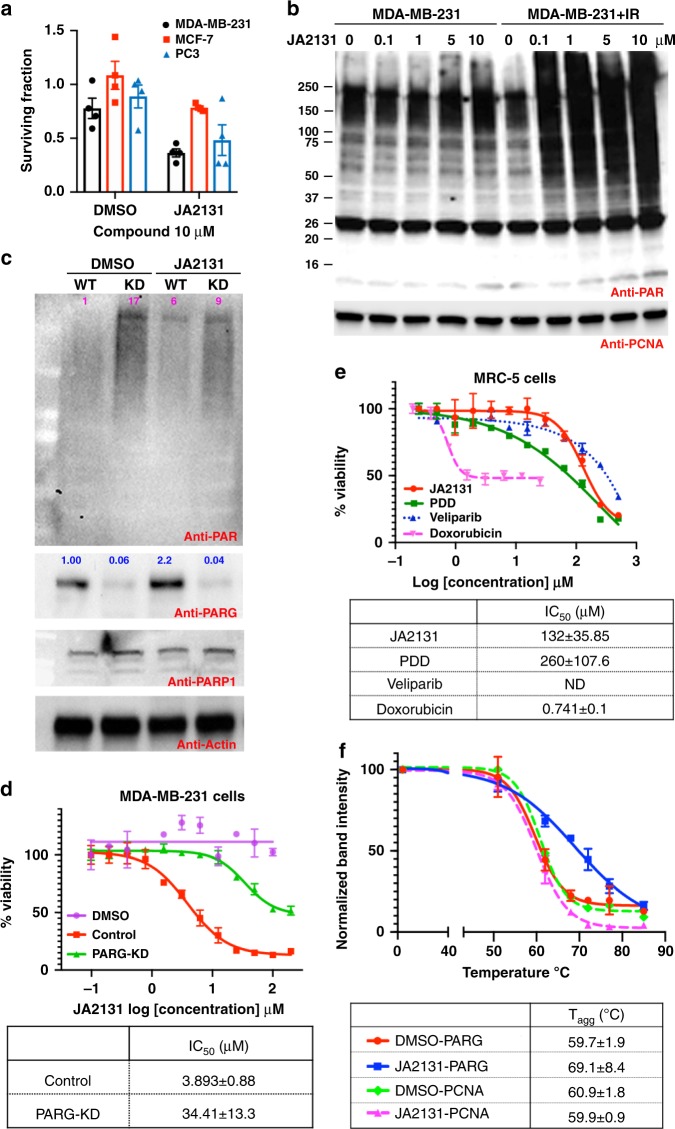
Selective inhibition of PARG by xanthine derivative JA2131. **a** Clonogenic survival assays of PC3, MDA-MB-231, and MCF-7 cells treated with PARGi. Cells were treated with either DMSO or 10 µM JA2131 for 2 h, irradiated with 3 Gy (MDA-MB-231 and MCF-7), 7 Gy (PC3) IR and grown for ~2 weeks, then colonies were fixed with methanol and stained with crystal violet. The results of three independent experiments are shown. Error bars = the standard error of the mean (SEM). **b** dose-response of JA2131 in MDA-MB-231 cells with and without IR. Cells were treated with designated concentrations of JA2131 for 2 h and then either left untreated or exposed to 3 Gy IR and allowed to recover for 1 h before lysis. Total protein was immunoblotted with anti-PAR antibody (upper panel), stripped and reprobed with anti-PCNA as a loading control. **c** MDA-MB-231 cells with stable scrambled shRNA (WT) or stable PARG shRNA, knockdown (KD), were treated with 5 μM JA2131 for 2 h, lysed and then total lysates were immunoblotted for anti-PAR (upper panel) followed by anti-PARG (upper middle panel), anti-PARP1 (lower middle panel) and anti-actin (bottom panel) antibody. Numbers on the blot shows relative band intensities determined by ImageJ. **d** The SRB cytotoxicity assay to evaluate JA2131 in MDA-MB-231 cells. Stable cells with scramble shRNA (control) and shPARG (PARG-KD) cells were treated with an increasing concentration of JA2131 for growth inhibitory IC_50_ determination. The data was normalized against 100% survival at the lowest inhibitor concentration. Graphs for IC_50_ were fitted to the four-parameter logistic equation using Prism8 and shown in the table (see methods). Error bars show the percent coefficient of variation. Equivalent concentrations of DMSO were used as a negative control. **e** The cytotoxicity of JA2131 along with PDD, Veliparib and Doxorubicin against MRC-5 cells. The IC_50_ curves were generated as described above and shown in the table. Error bars show the percent coefficient of variation. **f** CETSA for in-cell JA2131 target engagement. Normalized anti-PARG western blot intensities of three independent experiments were fitted as above and the estimated aggregation temperature (T_agg_) is shown in the tabulated insert. Source Data are provided as a Source Data file.

To determine the radiation sensitization effect of PARGi, a clonogenic cell survival assay was used to measure radiation sensitization in PC3, MDA-MB-231, and MCF-7 cell lines treated with JA2131. First, we defined the radiation dose response and the optimum cell plating number for each cell-line (Supplementary Fig. 7). Secondly, DMSO and the PARPi Olaparib were used as a negative and positive control respectively (Supplementary Fig. 8). The results show that PARG inhibitor JA2131 inhibits colony formation in all three cell lines. MCF-7 cells were less sensitive to JA2131 than the PC3 cells. The triple-negative breast cancer cells MDA-MB-231 were the most sensitive among the three cell-lines treated with JA2131 (Fig. 6a). Interestingly, in MCF-7 cells with the highest level of cytoplasmic PARG showed greatest sensitivity to the commercially available PARGi PDD00017273 (PDD herein) (Supplementary Fig. 8). These data suggest that underlying genetic variations that dictate PARG protein expression patterns and signaling could play an important role in the effectiveness of PARGi with implications for vetting future PARGi patient groups. In addition, we tested the effect of sustained JA2131 treatment alone or in combination with IR in colony formation. Indeed, JA2131 alone was sufficient to inhibit PC3 survival, but when combined with IR was more effective in reducing the number of surviving cell-colonies (Supplementary Fig. 9).

The triple-negative breast cancer cells MDA-MB-231 showed the largest response in our clonogenic survival assay (Fig. 6a). Thus, dose-dependent JA2131-induced cellular PARylation with and without IR was investigated. For combined IR treatment, MDA-MB-231 cells were treated with JA2131 for 2 h, followed by 7 Gy X-ray and then allowed to recover for an hour before cellular extraction and western blotting analysis. PARGi-treated cells alone showed a dose-dependent increase in cellular PARylation between 1 μM and 5 μM doses. However, PARGi combined with IR induced a massive increase in cellular PARylation at 100 nM (Fig. 6b). Notably, cells treated with radiation alone (Fig. 6b, lane 1) or inhibitor alone (Fig. 6b, lanes 2–5) did not show the level of PARylation observed when both treatments were combined, (Fig. 6b, lane 7–10) suggesting a synergistic, rather than an additive effect.

Using shRNA, next, we generated a stable MDA-MB-231 PARG knockdown (PARG-KD) cell-line (Supplementary Fig. 10). We compared PARGi JA2131-induced PARylation in PARG-KD and control cells. We reasoned that *PARG*-KD cells would show a reduced response to a selective PARGi as the stable PARG-KD cells would have to adopt a PARG-independent survival mechanism. MDA-MB-231 control cells and PARG-KD cells were treated with JA2131, then total cell lysates were subjected to immunoblotting with anti-PAR antibody, followed by immunodetection for anti-PARG, PARP1 and Actin antibody (Fig. 6c). As expected, untreated PARG-KD cells showed a higher baseline level of PARylation than the control cells. Treatment with JA2131 did not affect the level of PAR accumulation in PARG-KD cells; whereas, a measurable increase in PAR was seen in the MDA-MB-231 cells. Treatment of the cells under the same inhibitor regime with IR showed equivalent results (see Supplementary Fig. 11). Dose-response curves comparing control and PARG-KD cells further validate the selective action of JA2131 on control cells expressing PARG, but not on the PARG-KD cells (Supplementary Fig. 12). As a comparison, we also tested the inhibitor PDD, which induced PARylation in both control and PARG-KD cells. However, both cell-lines responded with increased PARylation from the PDD inhibitor, suggesting potential non-selectivity towards other cellular glycohydrolase (Supplementary Fig. 13).

To further evaluate the selectivity of JA2131, we performed Sulforhodamine B (SRB)^43^ cytotoxicity tests in MDA-MB-231 control and PARG-KD cells (Fig. 6d). The results show approximately a 10-fold higher IC_50_ value for PARG-depleted cells, compared to control cells with a normal level of PARG (see Fig. 6d, IC_50_ table). Notably, only 50% of the PARG-KD cells responded to the PARGi to produce the indicated IC_50_. In addition, we used normal human lung fibroblast cell-line MRC-5 to determine whether JA2131 exhibits specificity towards cancer cells. Our results show that the cytotoxic dose of JA2131 in MRC-5 cells is an order of magnitude higher than that seen for MDA-MB-231 cells (Fig. 6e, IC_50_ table). Doxorubicin and PARPi Veliparib were used as a positive and negative control, respectively. Thus, our collective cytotoxicity data show that JA2131 indeed acts through inhibition of PARG and that it selectively kills cancer cells. To further validate the inhibition of PARG in cells by JA2131, we performed the cellular thermal shift assay (CETSA)^44^ on PC3 cells treated with either DMSO or JA2131. The results show JA2131 treatment induced a 9 °C stabilization of PARG proteins in cells (Fig. 6f and Supplementary Fig. 14); whereas, the PCNA control showed no significant change in thermal stability in response to JA2131 treatment (see Fig. 6f).

We also evaluated JA2131’s specificity in vitro as an inhibitor of purified hPARG in the presence and absence of detergent (Triton-X 100, 0.1%) or a nonspecific target protein (bovine serum albumin BSA, 0.1 mg/ml). This concentration of Triton-X 100 is ~10-fold higher than its critical micelle concentration (CMC, 0.0155%), so it can effectively distinguish promiscuous and aggregating compounds^45^. Neither the addition of Triton-X 100 nor BSA significantly altered the inhibitory potency of JA2131 (Supplementary Fig. 15a). In addition, we also examined whether or not JA2131 could nonspecifically interfere with PARP1 activation. PARP1 is an abundant nuclear enzyme and PARylated PARP1 is an abundant substrate for PARG, following DNA damage. JA2131 did not inhibit PARP1 activation up to 100 μM; whereas, a potent PARP inhibitor Olaparib completely shuts down PAR synthesis by PARP1 (Supplementary Fig. 15b). These results, together with the observed extensive physical interactions between JA2131 and its binding site on hPARG (Fig. 3), support the conclusion that JA2131 selectively inhibits PARG activity.

### JA2131 causes replication fork defects

**Fig. 7:**
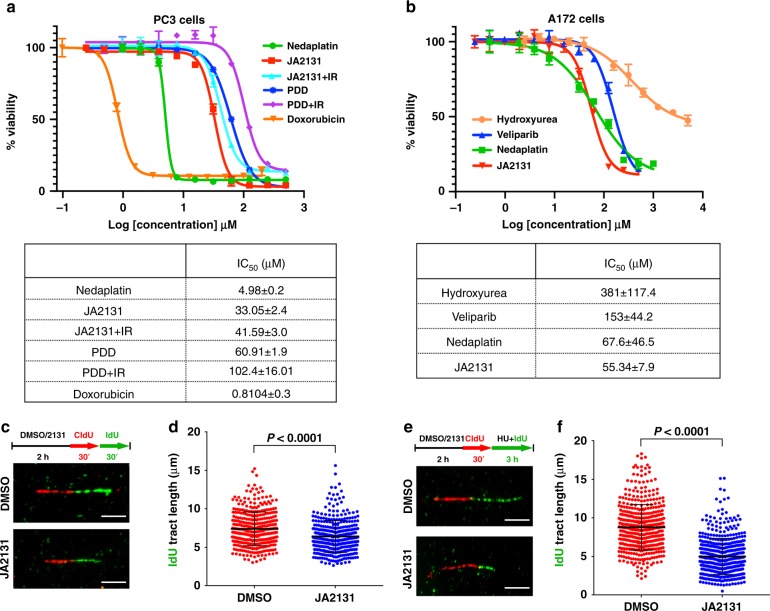
Cytotoxicity and fork stalling induced by PARGi. **a** Cytotoxicity of PC3 cells for the indicated compounds with or without 7 Gy IR for 72 h treatment periods. Normalized % viabilities are plotted against log concentration. Error bars show the percent coefficient of variation. The data table shows IC_50_ values for each compound. **b** Comparisons of the relative cytotoxicity of JA2131 against the designated DNA damaging agents for A172 glioblastoma cells. Treatment period 72 h. **c** A schematic of the dual-labeling regime used for the replication fork progression DNA fiber assay and representative DNA fiber images of the replication tract from DMSO and JA2131 treated HeLa cells. Scale bar 5 μm. **d** Quantification of the IdU tract analysis for more than 380 replication forks. **e** Schematic representation of HU (0.4 μM) treatment for fork progression analysis and the representative DNA fiber image of replication tracts of DMSO and JA2131 treated HeLa cells. Scale bar 5 μm. **f** Quantification of the IdU track of more than 470 replication forks for each data group. Mann-Whitney test was used to generate the *p*-value. The DNA spread assay is the representation of two independently repeated experiments. Source Data are provided as a Source Data file.

In the BRCA2-negative background, the depletion of PARG inhibits cell growth^27^. Yet, we find that PC3 cells with intact BRCA2 show exquisite DNA damage induction by JA2131 as seen by γH2AX foci formation (Fig. 5) and poor long-term survival (Fig. 6a). Therefore, we investigated if this JA2131-induced massive γH2AX foci formation leads to growth arrest and cell death. JA2131-induced cellular cytotoxicity in PC3 cells with or without IR, along with commonly used DNA damaging agents and the chemotherapeutic agents Doxorubicin and Nedaplatin (Fig. 7a, inset table). Surprisingly, there was no added benefit for combining PARGi with IR, in fact, cells actually survived slightly better in this 72 h assay period (see Fig. 7a, IC_50_ table). This is contrary to long-term clonogenic survival (Fig. 6a) and massive γH2AX foci formation (Fig. 5c) observed with combined PARGi and IR treatment. Indeed, we also observed a similar phenomenon with Olaparib-treated PC3 cells, where IR induced higher IC_50_ values compared to the non-IR treated samples (see Supplementary Fig. 16). Therefore, we reasoned that IR exposure activates homology-directed repair (HDR), which counteracts PARGi effects in the short term. However, PARGi-treated dividing cells accumulate damage from replication stress over time that decreases long-term survival as observed in clonogenic assays.

Furthermore, we tested our inhibitors in A172 glioblastoma cells that are resistant to PARPi and platinum-based chemotherapeutic agents. Cellular cytotoxicity assay showed our PARGi was more effective than Veliparib, but comparable in potency to Nedaplatin (Fig. 7b, inset IC_50_ table), indicating another cancer where PARGi could play an important therapeutic solution.

Recently, PARG depletion was shown to cause recruitment of double-strand-break-repair factors to chromatin in the absence damage and to slow replication fork progression,^36^ consistent with PARG function in replication stress protection through the resolution of dysfunctional replication-structures. Because our PARGi effectively killed cancer cells in the absence of DNA damage (Fig. 6d, Fig. 7a, b), we reasoned that JA2131 could be acting at the replication fork. Therefore, we tested whether our PARGi could induce replication stress defects. HeLa cells pretreated with JA2131 or DMSO for 2 h were used to evaluate fork progression in DNA fiber assay (Fig. 7c, d). Indeed, PARGi alone was sufficient to induce a significant reduction in replication progression in the absence of genotoxic stress. Under these conditions, PARGi induced a statistically significant reduction in the average IdU tract from 7.5 to 6.0 μm. Thus, we tested replication progression in the presence of mild genotoxic stress delivered by a low-dose of hydroxyurea (Fig. 7e, f). Under this mild genotoxic stress-condition, we observed a reduction in the average IdU tract length to 5.0 μm. These findings show that our PARGi recapitulates reported replication defects caused by PARG depletion^36^, a finding that further validates JA2131 selective action against PARG.

## Discussion

Reversible PARylation at DNA breaks and replication forks enforce control of modular assemblies of dynamic complexes, enabling damage detection, signaling, compartmental localization, and repair^46,47^. The ability to control PARylation reversal by PAR glycohydrolases^48^ particularly PARG, which is the predominant enzyme that removes PAR chains at sites of DNA damage^12,49^ offers the potential to control uniquely susceptible damage responses in cancer cells. Yet, there is no PARGi in the clinic, and existing inhibitors show various limitations. A classic PARG inhibitor is Gallotannin (GT), a large naturally occurring polyphenol that exhibits inhibition of PARG with an IC_50_ below 5 μM^50^. GT retards DNA repair^51^, protects against H_2_O_2_-mediated cell death^50^, sensitizes cancer cells to Cisplatin^52^, and can specifically kill *BRCA*-deficient breast cancer cells^27^. However, concerns exist about GT’s possible nonspecific mode of action and membrane permeability, as well as its antioxidant activity^53^. Salicylanilide derivatives appear to be nonspecific PARG inhibitors^45^. Rhodanine-based PARG inhibitors (RBPIs) are selective PARG inhibitors in vitro with low micromolar potency, but their bioavailability and cellular activity remain questionable^45^. Quinazolinedione sulfonamide derivatives target the PARG active site; however, rapid clearance in vivo renders them unsuitable as therapeutic agents^54^. The recently reported small-molecule PARGi PDD^54^ has strong effects on cellular PAR metabolism. Yet, our tests with PARG-depleted cells suggest a nonspecific mode of PDD action in mammalian cells. Such concerns with existing inhibitors motivated the efforts presented here for the development of a pharmacologically viable and selective PARGi via chemical library screening and structure-based drug design to improve potency and selectivity. These efforts led to the identification of thioxanthine/methylxanthine derivatives as chemically tractable and reasonably potent pharmacophores for specific and cell-permeable chemical knockdown of PARG (Fig. 1), for testing PARG biological functions and for therapeutic potential. Notably, methylxanthine derivatives occur in many plants and are among the most broadly consumed of all pharmacologically active compounds^40^.

The active JA2-4 series compounds (JA2-4 and JA2131) identified here display multiple complementary features and specific interactions with the hPARG active site, consistent with the observed sub-micromolar IC_50_. These PARGi are small in comparison to most drugs, so they provide suitable prototypes that suggest sites for modifications to improve potency and pharmacokinetic properties. Our current structural data furthermore reveals the molecular bases of the specific and competitive PARG inhibition by thio-methylxanthine derivatives (Figs. 2, 3). From the protein-ligand interactions identified here, the 6´-thio-methylxanthine derivatives JA2-4 and JA2131 compete with the adenine moiety of PAR substrates for active site binding.

Moreover, our structures unveil how the hPARG Tyr clasp switches between two conformational states to specifically engage methylxanthine derivatives containing different functional groups. This structural plasticity of the Tyr clasp is a key feature accompanying the binding of the potent JA2-4 series inhibitors. The Tyr clasp is a unique feature of PARG that is absent in other PAR glycohydrolase macrodomains, such as ARH3^55^, suggesting why PARG-KD cells show specific PARG inhibition by JA2131 (Fig. 3). A similar strategy of anchored plasticity to induce changes by inhibitor binding, allowed the design of potent selective inhibitors of nitric oxide synthases^56^, supporting the value of this inhibitor type for PARG.

Importantly, identification and characterization of JA2131 as a selective PARGi enabled direct examination of PARG signaling and the nature of PARP1-PARG relationship in DNA damage responses in cells. Notably, PARP1 is both the main polymerase and acceptor of PAR in response to DNA damage. Our data reveal that PARGi trapped PARP1 in a PARylated form (Fig. 4b). DePARylated PARP1 showed a single step change in molecular weight that suggests a potent glycohydrolase with a fast PAR turnover rate for alleviating PARP inhibition imposed by autoPARylation at the DNA damage site. Indeed, this idea is consistent with the observed rapid PARG activity post DNA damage.

PARG inhibition as a therapeutic option is directly questioned by a report showing PARG downregulation as a mechanism for acquiring PARPi resistance in BRCA2 and p53-null mouse cell-line^33^. Therefore, we generated BRCA2 and p53 proficient PARPi-resistant SUM149 human triple-negative breast cancer cell lines and investigated PARG protein expression. Our results detected no observable change in PARG protein expression in these cells (see Supplementary Fig. 17). However, JA2131-induced PAR accumulation was comparable to those observed for PC3 cells (Supplementary Fig. 16 and Fig. 5a). These observations suggest that selective loss of PARG in PARPi resistance may strictly depend on the underlying genetic context. Indeed, the general importance of PARG in cancer progression is underscored by finding that catalytically active PARG leads to poor prognosis and survival, while expression of enzymatically-defective protein played no part in tumor growth^35^. This is consistent with our TCGA database analysis, where most tumors show higher PARG expression compared to matched, normal tissues (Fig. 1a) and supports the possible value of a PARGi for ongoing investigations. Indeed, recently another small molecule PARGi was shown to delay DNA repair, sensitize cancer cells to DNA damaging agents, and be effective against PARPi-resistant tumors^57^. These data are in concert with the results presented here, underscoring the potential importance of PARGi cancer therapeutic.

Overall, our results identify unique targetable structural features of PARG that allowed us to identify and optimize a pharmacologically useful specific inhibitor. This PARGi is selective, cell-permeable, prevents PAR removal by PARG, and kills cancer cells at a level similar to Olaparib. As such, it is expected to contribute to understanding the roles of PARG and PAR turnover in multiple aspects of DNA damage responses. PARG is a unique mammalian protein without paralogs, in contrast to the human PARP family consisting of 17 related enzymes^58^. Thus, PARGi merits testing as potentially producing fewer off-target effects than PARP inhibitors currently employed as cancer therapeutics. With these points in mind, we hope that JA2131 will enable many studies evaluating the potential value of specifically inhibiting PARG in various cancer therapeutic strategies.

## Methods

### Chemicals

All NSC compounds were obtained from the Developmental Therapeutics Program (DTP) repository at NCI/NIH. JA1-5 (JS-2088), JA5-8 (BAS 05169959), and JA5-9 (AO-476/12797006) were purchased from Ryan Scientific. JA2-9 (Z57032584), JA5-10 (EN300-63858), and JA5-11 (Z385453050) were purchased from Enamine. JA2-8 (JFD03560SC) was purchased from Maybridge, and JA5-7 (F3350-0573) was purchased from Life chemicals. All inhibitors were dissolved at 40 mM concentrations in DMSO. Hydroxyurea, Nedaplatin, Doxorubicin, Veliparib, and Olaparib were purchased from SelleckChem. PDD00017273 was from Tocris.

### Protein expression and purification

The catalytic domain of rat PARG (residues 385–972) was purified as described previously^18^. The human PARG (hPARG) catalytic domain (residues 389–976) was cloned in pET28a (Novagen) with an N-terminal His-tag and expressed in *E. coli* Rosetta cells. hPARG^389–976^ was purified by Ni-NTA (Qiagen) affinity chromatography, loaded onto a heparin column (GE Healthcare), and then eluted with a salt gradient (0–1 M NaCl). hPARG^389–976^ was further purified, using a Superdex 200 size-exclusion chromatography. For the preparation of PARylated PARP1, the DNA binding domain (DBD; residues 1–374) of human PARP1 and the PARP1C catalytic domain (residues 375–1014) were purified as described previously^18^. For crystallization, human PARG catalytic domain (residues 448–976) containing the six surface entropy reduction mutations^20^ was cloned in pET28a vector with an N-terminal His-tag and expressed in *E. coli* HMS174 cells expressing GroESL chaperon. The GST-tagged central BRCT domain of human XRCC1 (BRCT1, residues 294–417; cloned in pGEX-6p1) was expressed in *E. coli* Rosetta cells and purified by glutathione affinity chromatography. Following cleavage of the GST-tag with PreScission protease (GE Healthcare), the BRCT1 domain was purified using a Sephacryl 100 (GE Healthcare) size-exclusion column. For the biotinylation of BRCT1, the BRCT1 domain of XRCC1 was cloned in pGEX-6p1 with a C-terminal biotin acceptor peptide (BAP) tag and co-expressed with the BirA biotin ligase (pACYC184-BirA plasmid; Avidity) in *E. coli* BL21 (DE3) cells. The biotinylated BRCT1 was purified using the same protocol as the GST-BRCT1 protein.

### TR-FRET PAR turnover assay

For a high-throughput screening (HTS) of PARG inhibitors, we developed a high-throughput PARG activity assay using the TR-FRET system (a manuscript describing the details of this assay will be published elsewhere). Briefly, the fluorescein molecule (FITC) was enzymatically incorporated into PARP1 in a reaction containing PARP1C (2 μM), the PARP1 DBD (2 μM), a 24 mer nicked DNA oligo (2 μM), and a mixture of unlabeled NAD^+^ (Sigma) and FITC-NAD^+^ (Trevigen) substrates (total NAD^+^ concentration of 100 μM). After incubation for 1 h at 37 °C, PARylated PARP1 was desalted using a PD-10 desalting column (GE healthcare) in a buffer containing 25 mM Tris-HCl (pH 7.5), 50 mM NaCl, and 0.01% NP-40 and used as the FRET acceptor. The biotin-tagged BRCT1 domain of XRCC1 (BRCT1, 5 nM) was conjugated with an equimolar concentration of Tb^3+^-chelated streptavidin (Life Technologies) in TR-FRET assay buffer (FRET donor). To monitor dose-response inhibition of PARG activity (supplementary Table 2), rat PARG^385–972^ (12.5 nM) was pre-incubated with compounds for 1 h at room temperature. The reaction was started by the addition of the FRET pair of FITC-labeled PARP1 (42 nM) and Tb^3+^-BRCT1 (5 nM). The TR-FRET signal was subsequently monitored for 60 min.

### High-throughput screening of PARG inhibitors

Small molecule inhibitors of PARG were identified by high-throughput screening (HTS) using the TR-FRET PAR turnover assay. Compounds (final concentration of 6.7 μM) from the NCI Diversity Set II library (1990 compounds) were pre dispensed into black polystyrene 384-well plates (Corning) using a Hummingbird liquid handler (Digilab). Then, 10 μl of rat PARG^385–972^ (12.5 nM and 25 nM) were subsequently dispensed into plates, using a multi-drop combi nL reagent dispenser (Thermo Scientific) and incubated with compounds for 1 h at 4 °C. The reaction was initiated by the addition of 20 μl of the premixed FRET pair of FITC-labeled PARP1 (42 nM) and Tb^3+^-BRCT1 (5 nM), and the TR-FRET signal was monitored every 4 min for 40 min (11 data points). Hits were selected by visually comparing the rate of the TR-FRET signal decrease in each well to that of negative control reactions containing DMSO (Fig. 1b and see Text for details). The HTS was performed at the High-Throughput Screening Core (HTSC) at Washington University School of Medicine.

### Gel-based PARG activity assay

PARP1C (2 μM) is enzymatically auto-modified in a reaction containing the PARP1 DBD (2 μM), a 24 mer nicked DNA oligo (2 μM), and NAD^+^ (300 μM) for 1 h at 37 °C, as described previously^18^. Human PARG^389–976^ (1 nM) was pre-incubated with compounds for 1 h at room temperature. The reaction was initiated by the addition of PARylated PARP1 (final concentration of 500 nM) and quenched after 30 min incubation at room temperature by adding SDS-PAGE sample buffer. The gel was visualized by sypro-ruby staining^18^ and the retained PARylated PARP1C species were quantified using ImageJ^59^. PARylated PARP1C that migrates slower than unmodified PARP1C was quantified. Control reactions in the absence of PARG (100%) or compound (0%) were used for normalization of the degree of PARG inhibition by compounds (% inhibition). The IC_50_ values were determined by fitting the dose-response data for each compound to a four-parameter logistic equation using SigmaPlot (Systat Software Inc.).

To test the specificity of JA2131, human PARG^389–976^ (1 nM) was pre-incubated with JA2131 (5, 10, and 20 μM) in the presence and absence of 0.1% Triton-X 100 or 0.1 mg/ml BSA for 1 h at room temperature. The reaction was initiated, quenched, visualized, and quantified as described above.

### Crystallization of human PARG catalytic domain

Crystals of the unliganded hPARG^448–976^ were grown by hanging drop vapor diffusion as described previously^20^. hPARG^448–976^ (7.5 mg/ml) was mixed with an equal volume of well solution, containing 16–24% (w/v) PEG 3350, 0.1 M PCTP (0.04 M sodium propionate, 0.02 M sodium cacodylate, 0.04 M Bis-Tris propane) pH 7.5 and 0.15 M MgCl_2_ and incubated at 22 °C. To soak inhibitors, crystals were harvested in a cryoprotectant solution containing 26% PEG 3350, 0.05 M PCTP pH 7.5, 0.1 M NaCl, 0.15 M MgCl_2_, and 2.5% glycerol. Crystals were soaked with inhibitors at 5 or 10 mM concentrations for 15 h, and then were flash-cooled in liquid nitrogen. X-ray diffraction data for five structures were collected in-house at 100 K using a Rigaku MicroMax007 rotating anode X-ray generator equipped with Xenocs Mirrors and a MAR image plate, and processed using HKL2000^60^ and SCALEPACK^60,61^. All crystals have one hPARG molecule per asymmetric unit and diffracted to 1.7–2.0 Å resolution. X-ray diffraction data statistics are shown in Supplementary Table 3 of the online supplementary information. The structures of hPARG bound to the methylxanthine derivatives were determined by molecular replacement using Molrep of the CCP4i suite^62^ with the unliganded hPARG^448–976^ structure (PDB ID: 4B1G)^20^ as a search model. The crystallographic models for the hPARG-inhibitor complexes were constructed using COOT^63^ and refined using REFMAC^64^. Crystallographic data statistics are shown in Supplementary Table 3. All structural figures were prepared using PyMOL (www.pymol.org) or Chimera^65^.

To show reproducibility and confirm the in-house PARG-inhibitor structures, hPARG crystals were also prepared for synchrotron data collection. hPARG crystals were grown as described, soaked with 10 mM JA2131 or JA2120 compound for 1–2 h, then flash-cooled in liquid nitrogen. X-ray diffraction data were acquired at Stanford Synchrotron Radiation Lightsource (SSRL) beamline 9-2^66,67^. Data were processed with XDS to 1.6–1.7 Å^66,68^, solved by molecular replacement as described, and refined in Phenix^69^. Diffraction and crystallographic data statistics are shown in Supplementary Table 4 of the online supplementary information. PARG-inhibitor structures determined from synchrotron data were similar in resolution and conformation to complexes determined from in-house data despite having a different space group.

### Cell culture

PC3, MDA-MB-231 cells were maintained in RPMI 1640, MCF-7 cells in Eagle’s Minimum Essential Medium (EMEM), MRC-5 cells in Eagle’s Minimum Essential Medium and Hela and U2OS cells in Dulbecco’s modified Eagle’s high glucose medium (DMEM) supplemented with 10% (vol/vol) fetal bovine serum (FBS) and 1% antibiotic/antimycotic (Lonza) in a humidified incubator with 10% CO_2_. All cell lines were purchased from ATCC and regularly checked for mycoplasma and cell authentication. SUM 149PT cells were cultured in F-12 Hams (Gibco) supplemented with 5% fetal bovine serum, insulin, and hydrocortisone. Colony formation assays were carried out in 6-well plates, 500 cells were used per well and following designated treatment, and colonies were grown for 2 weeks. Cells were fixed with methanol (100%), stained with crystal violet and analyzed using a GelCount instrument (Oxford Optronix Ltd). Crystal Violet C6158 was purchased from Sigma. PARG knockdown cells were generated by infecting MDA-MB-231 cells with shPARG lentiviral particles (sc-106355-V, Santa Cruz Biotechnology), which contain a pool of three shRNA plasmids. After 24 h infection, 3 µg/ml puromycin (Invitrogen) was added and antibiotic-resistant cells were further expanded and used for experimentation. Sequence-1; hairpin sequence: GATCCGGAAACGGTACTCTACTAATTCAAGAGATTAGTAGAGTACCGTTTCCTTTTT, sense: GGAAACGGUACUCUACUAAtt, Antisense: UUAGUAGAGUACCGUUUCCtt. Sequence-2; hairpin sequence, GATCCGAAGGATGCTATTCTGAAATTCAAGAGATTTCAGAATAGCAT-CCTTCTTTTT, sense: GAAGGAUGCUAUUCUGAAAtt, antisense: UUUCAGAAUAGCAUCCUUCtt. Sequence-3; hairpin sequence: GATCCGGAAACCGGAGAAACTTAATTCAAGAGATTAAGTTTCTCC-GGTTTCCTTTTT, sense: GGAAACCGGAGAAACUUAAtt, antisense: UUAAGUUUCUCC-GGUUUCCtt. Control cells were generated using the same process except that scramble lentiviral particles with scrambled sequences were used (sc-108080).

### Cellular thermal shift assay (CETSA)

CETSA was performed as described by Molina et al.^44^. Briefly, PC3 cells were treated with 10 μM JA2131 or an equivalent volume of DMSO for 2 h, washed three times with PBS and extracted for total cell lysate. Ten percent glycerol was added to the samples before subjecting to heat. Gradient thermal cycler (C1000 Touch, Bio-Rad) was used to heat samples at 42, 57, 66, 72, 77, and 85 °C for 3 min and chilled on ice for 5 min. Then, samples were centrifuged at 14,000 rpm for 20 min at 4 °C. Supernatants were carefully removed and analyzed by western blotting analysis.

### Antibodies and western blots

Cells were grown in 6-well plates and 10 cm dishes, treated with PARGi for 2 h, irradiated and recovered for 1 h before harvesting. Cells were irradiated, ranging from 1 to 10 Gy using RAD SOURCE, RS-2000, Biological System, RadSource.com. Cells were washed with phosphate buffer and saline (PBS) and lysed with buffer containing 50 mM Hepes, pH 7.5, 1% (vol/vol) igepal-C630, 1 mg/ml bacitracin, 1 mM EDTA, 10 mM NaF, 1 mM sodium orthovanadate, 10% (vol/vol) glycerol, 50 mM NaCl, 1 mM PMSF, and Protease Inhibitor Cocktail Set III (EMD Millipore). The detergent-soluble fraction was used for western blotting. Cell fractionation was carried out using the Subcellular Protein Fractionation Kit, (#78840, ThermoFisher) according to the manufacturer’s instruction. Primary antibodies were purchased from the following sources; Actin, GAPDH, γH2AX and Histone-H3 was from Cell Signaling Technology. H2AX and LAMB1 were from Invitrogen/ThermoFisher. PCNA and p53 were from Santa Cruz Biotechnology. Anti-PAR (10 H) was from EMD Millipore and anti-PARG was from or Cell Signaling Technology. Anti-PARP1 from Abcam. Secondary antibodies were conjugated HRP (Cell Signaling) and ECL Clarity was purchased from Bio-Rad. All primary and secondary antibodies were diluted 1:1000 with 5% non-fat Blotting Grade Blocking Reagent in 1x TBST, except for anti-PAR 1:500. Uncropped western data are presented in the Supplementary Fig. 18.

### Immuno-staining and imaging

PC3 cells were grown to 75% confluence in 24-well 25 μm film bottom Eppendorf black cell Imaging plates (0030741005), treated with either DMSO or designated inhibitors for 2 h, irradiated 7 Gy X-ray and allowed to recover for an hour before fixing with 4% (wt/vol) paraformaldehyde, pH 8.0. All subsequent steps were performed at pH8.0. Cells were washed 6–7 times with PBS, permeabilized 0.5% Triton X-100 in PBS on ice for 20 min, blocked overnight with PBS/5% Goat serum/3%BSA/0.5% Triton X-100. Cells were incubated with primary antibody 1:250 dilution in PBS/5%BSA/0.5% Triton X-100 overnight at 4 °C. Following 6× wash with PBS pH 8.0, cells were exposed to fluorescence-labeled secondary antibody diluted 1:500 in PBS/5%BSA/0.5% Triton X-100 for 1 h at room temperature. Following 6–7× PBS washes, cells were fixed again with 4% PFA at room temperature for 20 min. After a further 6× PBS washes cells were imaged in PBS buffer using ImageXpress high-content imager (Molecular Devices Inc). Statistical analysis was performed with MetaXpress Software.

### Sulforhodamine B (SRB) cytotoxicity assay

Cells were plated in 96-well plates, treated as designated in reduced 5% FBS and fixed with trichloroacetic acid after 72 h, as previously described by^43^. PARG inhibitor JA2131 was titrated typically from 300 to 0.15 μM. Colorimetric or fluorescence analysis was performed in a FlexStation 3, using SoftMax Pro 7.0, Molecular Devices. Normalized graphs were generated with Prism 8 software, non-linear 4-parameter data fitting was performed for IC_50_ calculation.

### DNA fiber assay

DNA fiber assay was conducted as described previously (Castillo et al. 2014). Briefly, cells pretreated with JA2131 (10 µM) or DMSO for 2 h were labeled with 100 µM CldU for 30 min and exposed to 250 µM IdU (with or without 0.4 mM hydroxyurea treatment) for another 30 min or 3 h. After labeling and treatment, cells were lysed and DNA fibers stretched onto glass slides. Immunofluorescence was carried out using α-IdU/α-BrdU (BD Biosciences) and α-CldU/α-BrdU (Abcam) and secondary antibodies, including Alexa Fluor 488 (green) and Alexa Fluor 555 (Invitrogen). Images were taken using a Nikon 80 microscope and analyzed using ImageJ software. Statistics were calculated using Prism software. 5-Chloro-2’-deoxyuridine (CIdU) and 5-Iodo-2’-deoxyuridine (IdU) were from Sigma.

### TCGA analysis

We used the TCGA-Assembler suite^70^ with the Assay Platform option set to gene to obtain the normalized Rsem RNA-seq gene expression data from TCGA (https://cancergenome.nih.gov) project. Data were processed with in-house scripts (C++ and Bash) and plotted with the R package “ggpllot2” and “ggpubr”.

### Reporting summary

Further information on research design is available in the Nature Research Reporting Summary linked to this article.

## Supplementary information

Supplementary Information

Reporting Summary

## Data Availability

The data supporting the findings of this manuscript are available from the corresponding author upon reasonable request. Coordinates and structure-factor files for human PARG complexed with JA2-4, JA2-8, JA2-9, JA2120, and JA2131 have been deposited in the Protein Data Bank, with accession codes; 6O9X, 6O9Y, 6OA0, 6OA1, and 6OA3.

## Source data

Source Data
