# Supplementary material for: Selective small molecule PARG inhibitor causes replication fork stalling and cancer cell death
Source: Nat Commun. 2019 Dec 11;10:5654. doi: 10.1038/s41467-019-13508-4 (PMC6906431; doi:10.1038/s41467-019-13508-4)

Supplementary Information

Selective small molecule PARG inhibitor causes replication fork stalling and cancer cell death

Houl et al.

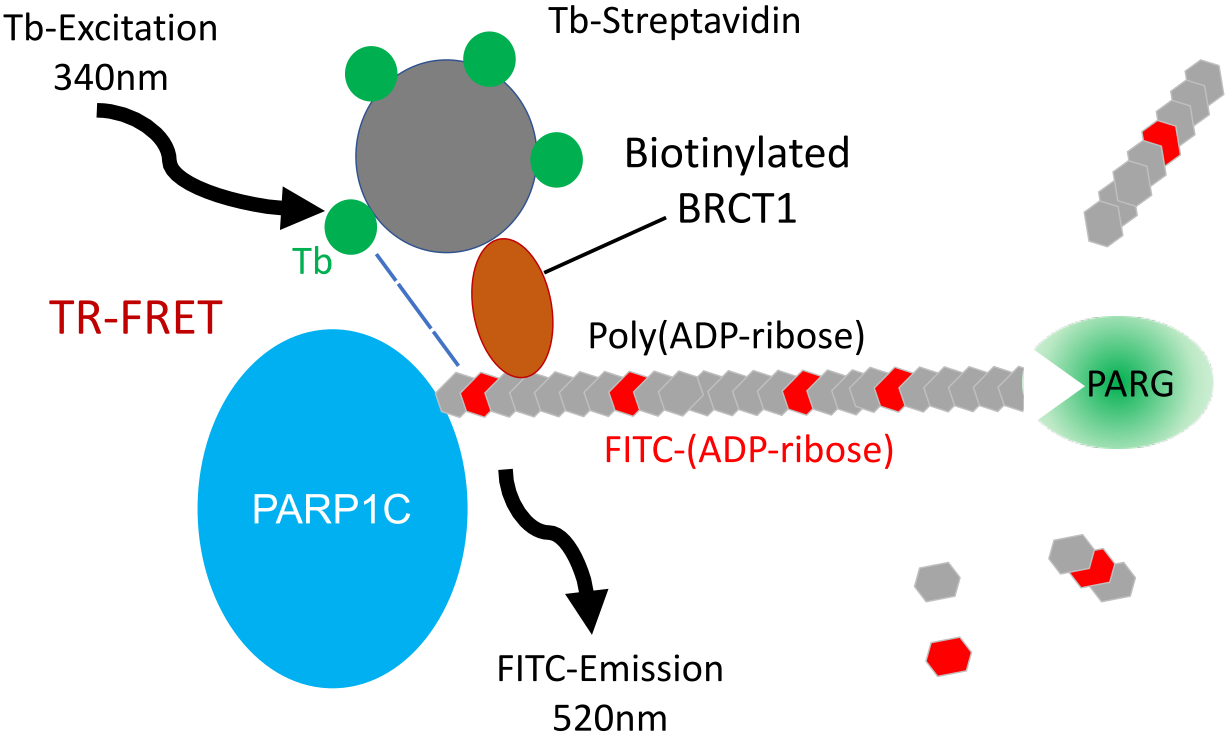

Supplementary Fig. 1: A high-throughput TR-FRET assay for PAR turnover. Fluorescein (FITC) molecules are incorporated into PAR chains during enzymatic PARylation of PARP1C (see Supplementary Methods). FITC-labeled PARP1C (FRET acceptor) is bound to the biotinylated BRCT1 domain of XRCC1 that is complexed with a Tb^{3+}-streptavidin conjugate (FRET donor). The TR-FRET intensity ratio (F_{520}/F_{495}) resulting from PARP1-XRCC1 complex formation is > 10-fold higher than the background signal when the FITC-PARP1 acceptor or the BRCT1 domain is omitted from the reaction. Addition of PARG to the labeled PARP1-XRCC1 complex removes the PAR posttranslational modification from PARP1, causing the complex to fall apart with loss of the TR-FRET signal (Fig. 1b). For full detail see Kim et. al.,2015 A quantitative assay reveals ligand specificity of the DNA scaffold repair protein XRCC1 and efficient disassembly of complexes of XRCC1 and the poly(ADP-ribose) polymerase 1 by poly(ADP-ribose) glycohydrolase. Republished with permission of American Soc for Biochemistry & Molecular Biology, A quantitative assay reveals ligand specificity of the DNA scaffold repair protein XRCC1 and efficient disassembly of complexes of XRCC1 and the poly(ADP-ribose) polymerase 1 by poly(ADP-ribose) glycohydrolase, Kim et al., J Biol Chem 290, 3775-3783 (2015); permission conveyed through Copyright Clearance Center, Inc.

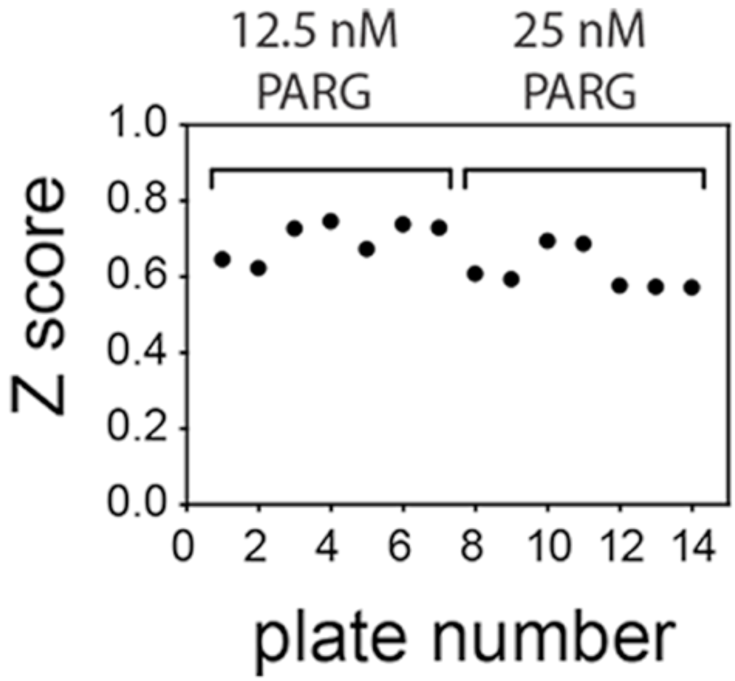

Supplementary Fig. 2: Z-score showing the accuracy and reproducibility of the screen.

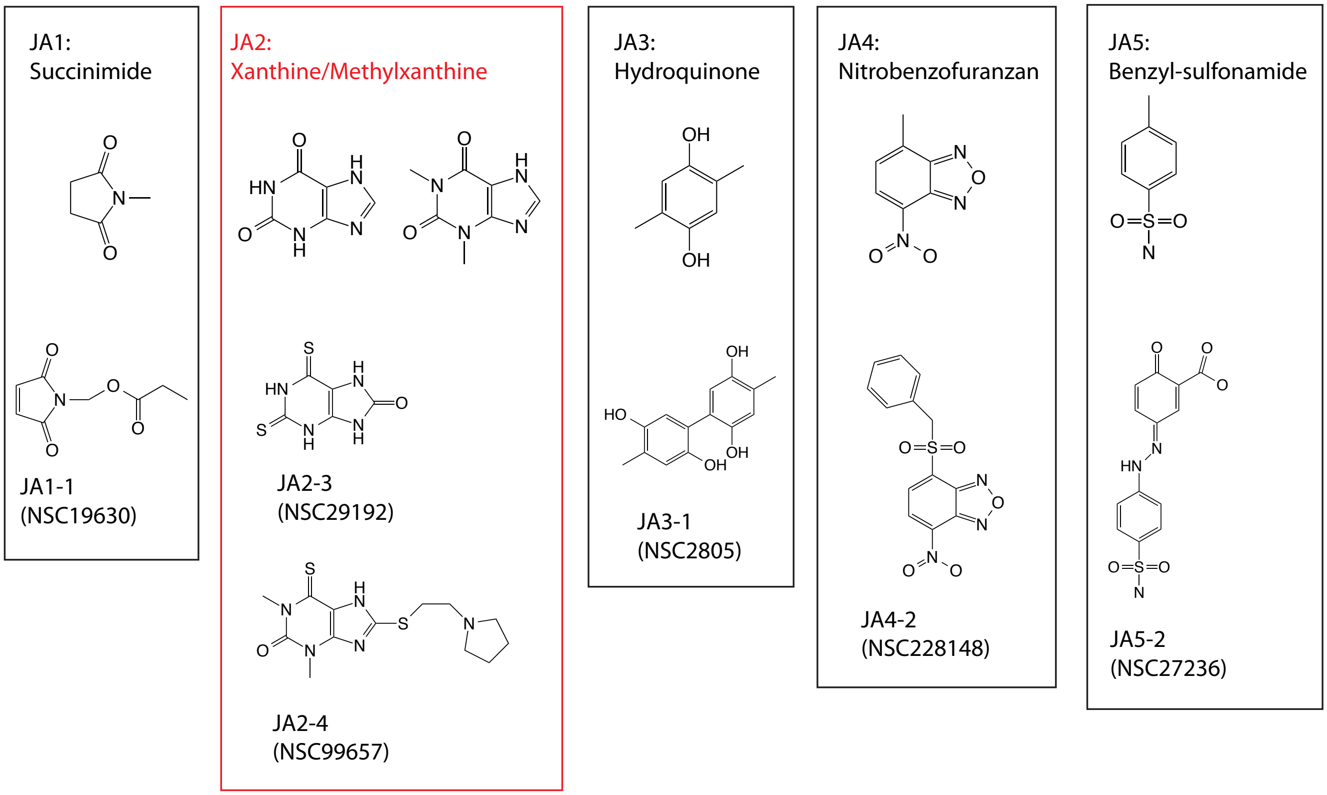

Supplementary Fig. 3: Five main chemotypes identified from the high-throughput screening (HTS). A total of 51 hits were identified from the HTS, and subsequently grouped into five chemotypes (JA1, JA2, JA3, JA4, and JA5). Representative HTS hits for each scaffold are shown. All 51 initial hits selected from the HTS are listed in Supplementary Table 1. Among five chemotypes, xanthine/methylxanthine (JA2) was selected as the lead pharmacophore for further analysis and optimization, based on the structural similarities to adenine, potency *in vitro*, and potential bioavailability.

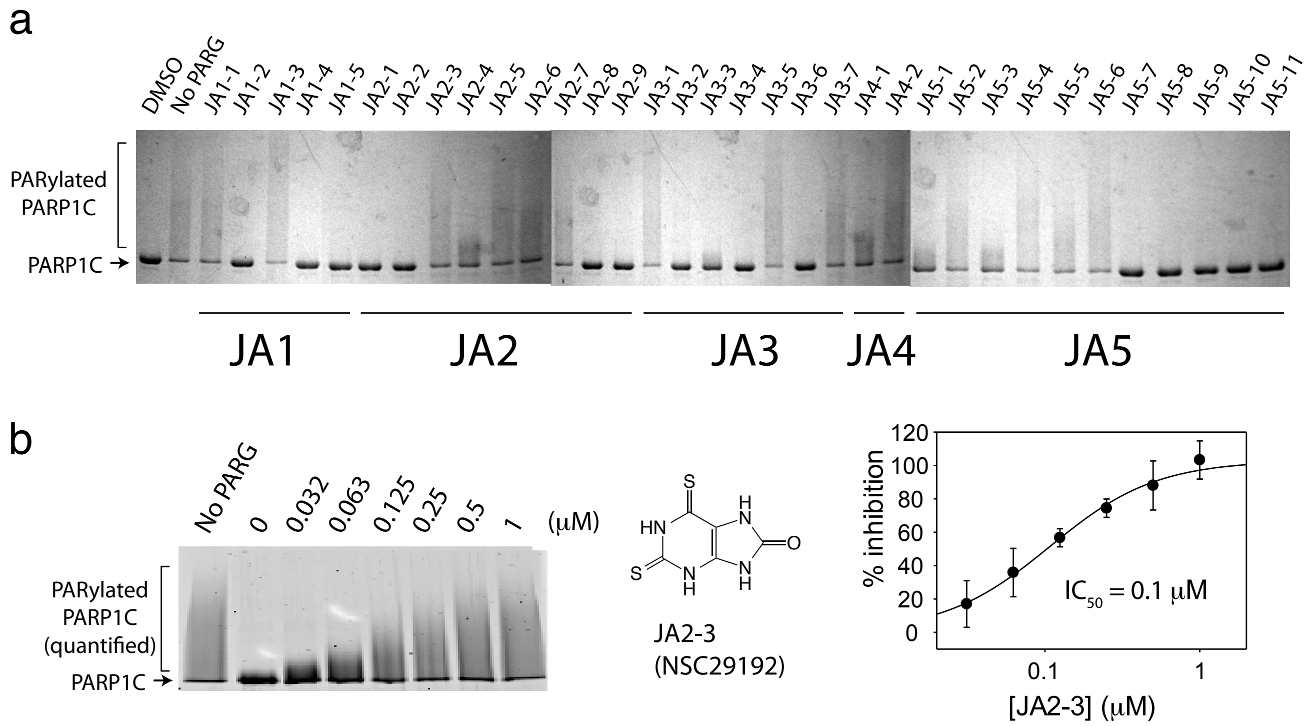

Supplementary Fig. 4: Structure-activity relationships (SAR) of compounds from five main chemotypes. (a) A total of 34 representative HTS hits and commercially available analogues from each chemotype (Fig. S3 and Supplementary Table 2) were analyzed using the gel-based PARG activity assay. This gel-based assay directly monitors the change of PARylation status of PARP1 as a substrate of PARG. Candidate inhibitors (25 μM) were preincubated with rat PARG (12.5 nM) for 1 hour at room temperature, and the reaction was started by addition of PARylated PARP1 (500 nM). The reaction was quenched after a 30-minute incubation at room temperature by adding SDS-PAGE sample buffer. PARylated PARP1C runs as a smear on SDS-PAGE that is reduced to a single band by PARG treatment. These results are in good agreement with IC_{50} values estimated from dose-response studies using the TR-FRET assay (Supplementary Table 2). (b) Quantification of the dose-response PARG inhibition by JA2-3 using the gel-based PARG activity assay. hPARG (1 nM) was preincubated with increasing concentrations (0.032 – 1 μM) of JA2-3 for 1 hour at room temperature before PARylated PARP1C (500 nM) was added. The reaction was quenched after a 30-minute incubation at room temperature. PARylated PARP1C migrates slower than unmodified PARP1C and was quantified using ImageJ. To determine the IC_{50} value, the dose-response data were plotted as a function of JA2-3 concentration and fitted to the four-parameter logistic equation using SigmaPlot. The rate of the PARG-dependent signal loss in the TR-FRET PAR turnover assay is not equivalent to the steady-state rate of PAR turnover, because the TR-FRET assay can't detect signal loss until the length of PAR chains become shorter than 7 ADP-ribose units. Therefore, we used the gel-based PARG activity assay to accurately determine the IC_{50} values throughout the manuscript.

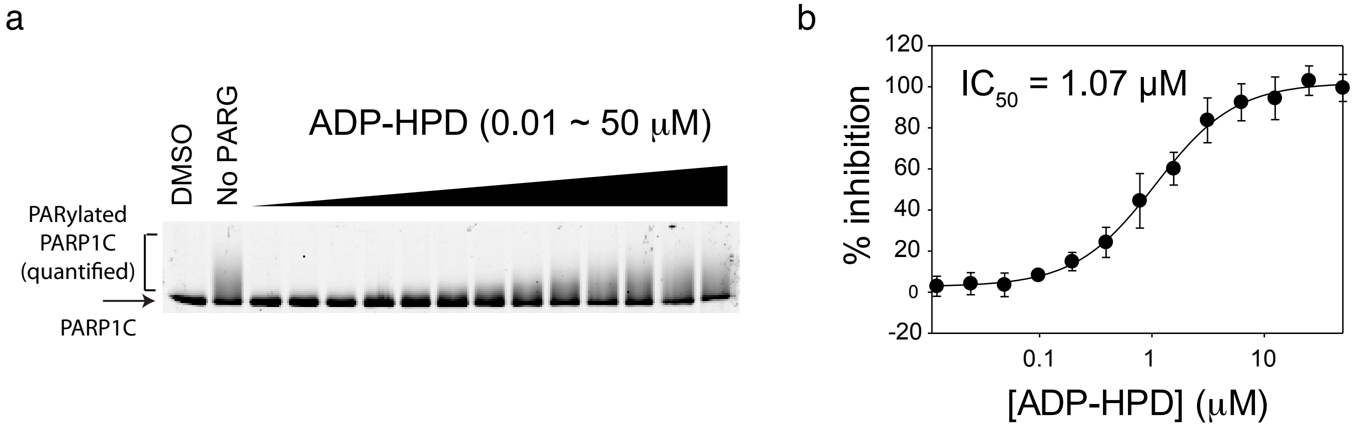

Supplementary Fig. 5: Inhibition of PARG activity by ADP-HDP. (a) Dose dependent inhibition of PARG by ADP-HDP. (b) Quantification of the dose-response PARG inhibition by JA2-3 using the gel-based PARG activity assay. hPARG (1 nM) was preincubated with increasing concentrations (0.032 – 1 μM) of JA2-3 for 1 hour at room temperature before PARylated PARP1C (500 nM) was added. The reaction was quenched after a 30-minute incubation at room temperature. PARylated PARP1C migrates slower than unmodified PARP1C and was quantified using ImageJ. To determine the IC_{50} value, the dose-response data were plotted as a function of JA2-3 concentration and fitted to the four-parameter logistic equation using SigmaPlot. The rate of the PARG-dependent signal loss in the TR-FRET PAR turnover assay is not equivalent to the steady-state rate of PAR turnover because the TR-FRET assay can't detect signal loss until the length of PAR chains become shorter than 7 ADP-ribose units. Therefore, we used the gel-based PARG activity assay to accurately determine the IC_{50} values throughout the manuscript.

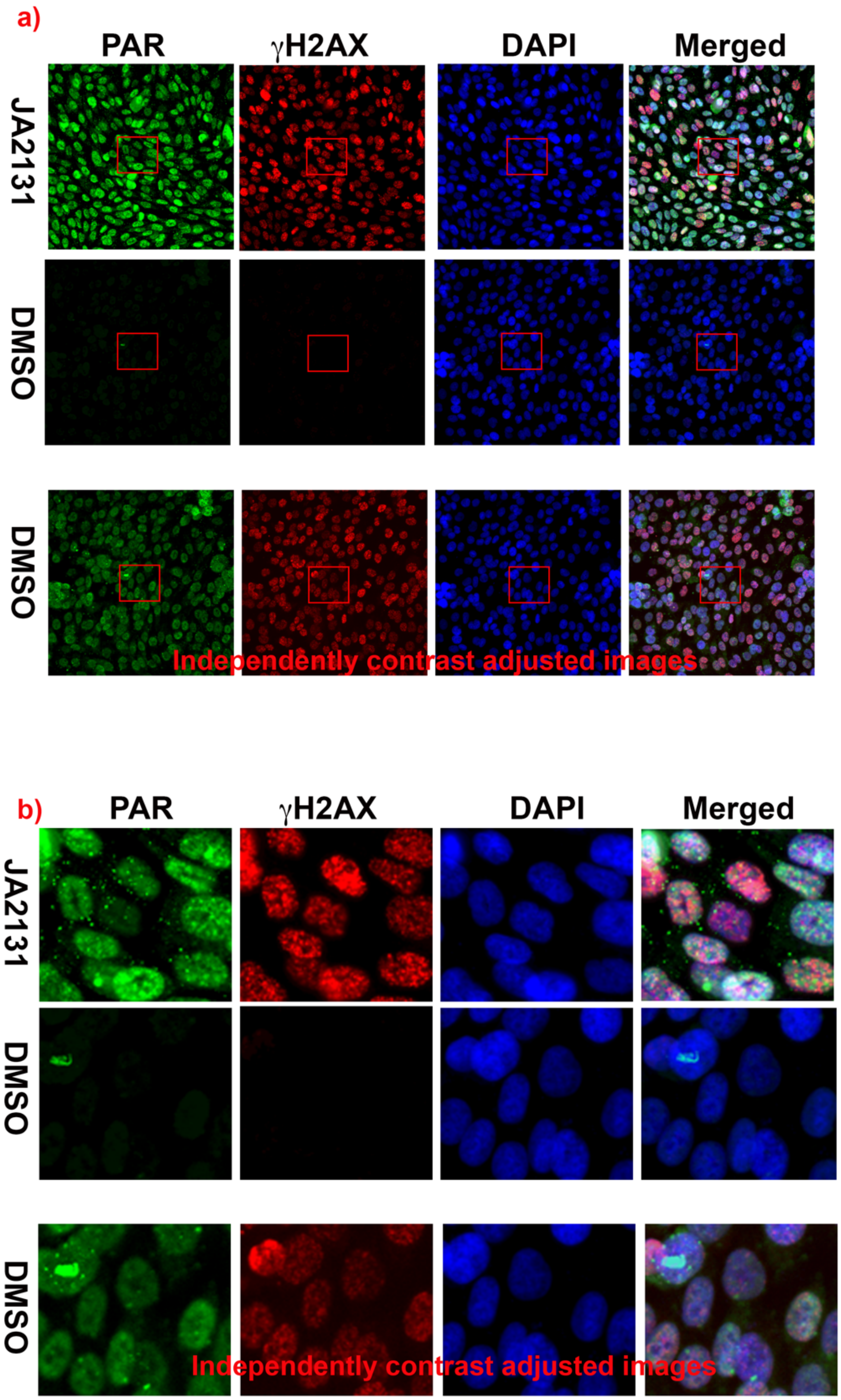

Supplementary Fig. 6: Magnified images that were used for quantitative analysis. (a) Magnified 1x1 image of the denoted * of the 3x3 data on Fig. 5a. The quantitative image with matched contrast with JA2131 and independently contrast adjusted images for DMSO is shown below (b) for the second magnification of the image marked with red box on (a). As above, an independently contrast adjusted DMSO image shown in the bottom.

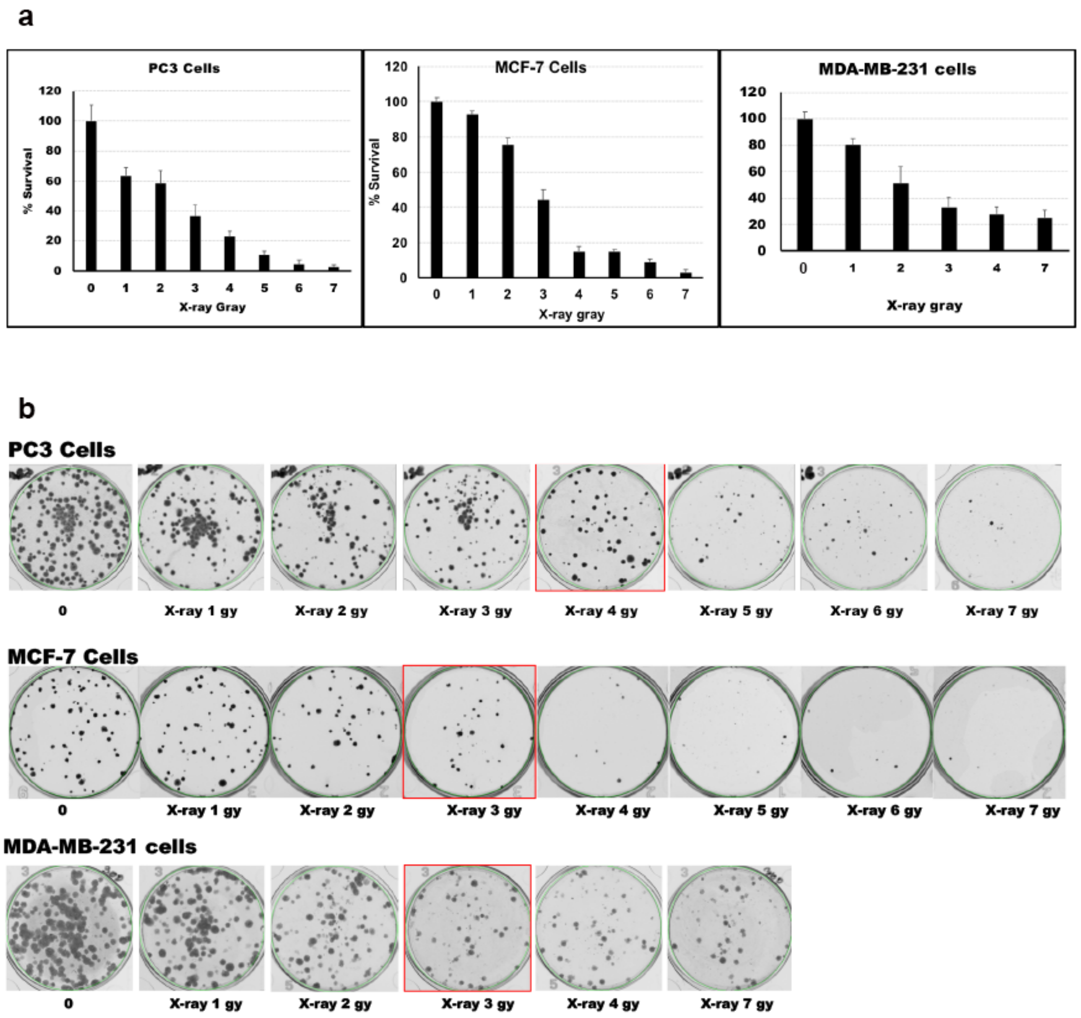

Supplementary Fig. 7: Clonogenic assay to determine the IC_{50} value for X-ray dosage for PC3, MCF-7 and MDA-MB-231 cell-lines. (a) Dose response survival curve obtained from counting colonies from three independent. (b) Representation of raw data used for obtaining the dose response curve in ‘a’. Red box indicates the mid-range of the dose for each cell type.

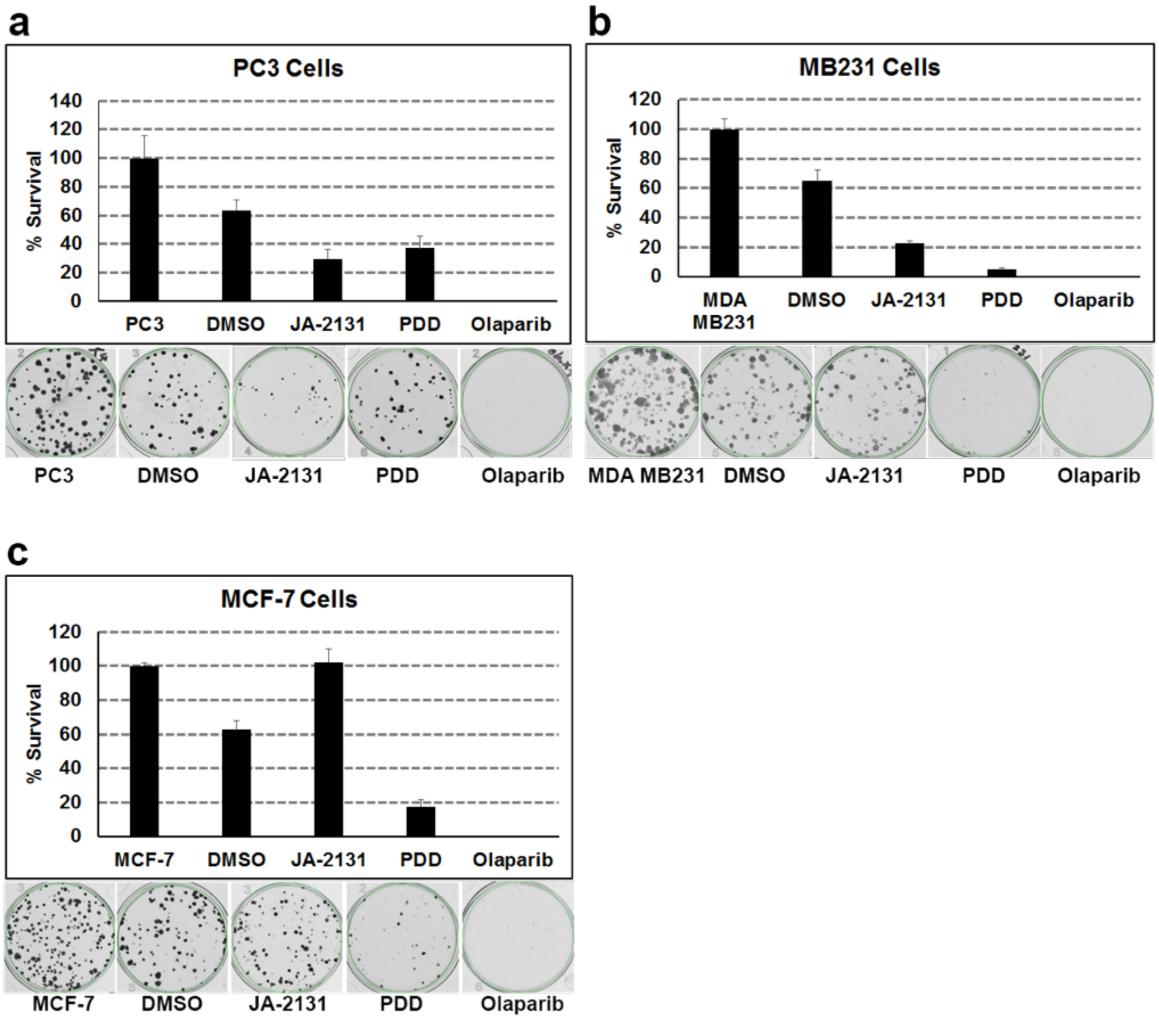

Supplementary Fig. 8: Clonogenic survival of cells in the presence of PARGi and PARPi (a) Clonogenic survival of PC3 cells in response to indicated compounds. (b) Clonogenic survival of MDA-MB-231 cells in response to indicated compounds. (c) Clonogenic survival of MCF-7 cells in response to indicated compounds. Olaparib and DMSO was used as a positive and negative control respectively. Cells were treated with 10 μM indicated compounds for 1 hour followed by 4 Gy IR and allowed to recover for 14 days. The surviving colonies were fixed with methanol, stained with crystal violet and analyzed using an automated colony counter, GelCount instrument (Oxford Optronix Ltd).

a)

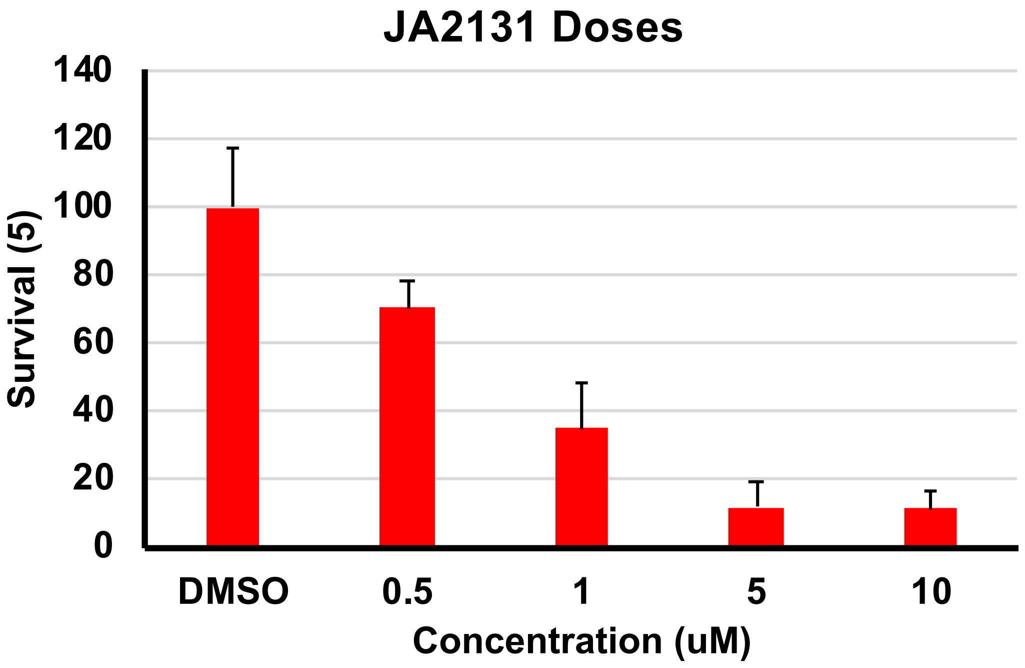

b)

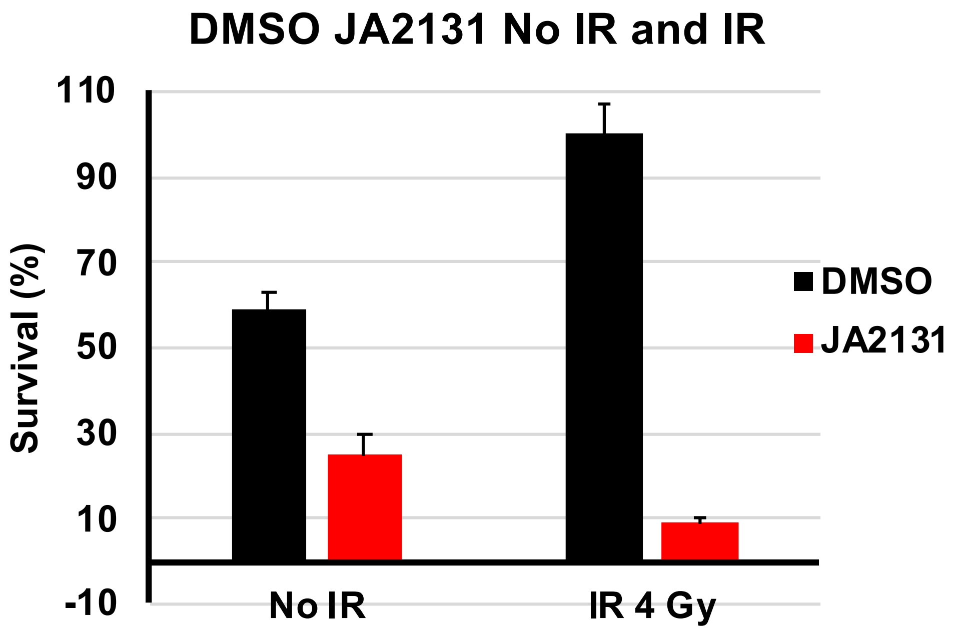

Supplementary Fig. 9: Inhibition of cell-growth by JA2131. (a) Dose-dependent PC3 cells survival in the presence of titrating concentration of JA2131. (b) PC3 cells treated with 10 μM JA2131 or equivalent DMSO then either irradiated with 4 Gy IR or left untreated. Following two weeks surviving colonies were counted and presented.

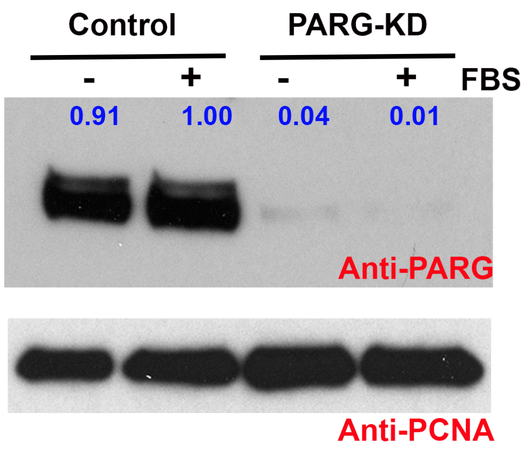

Supplementary Fig. 10: Validation of PARG knockdown cells in MDA-MB-231 cells. Cells in normal growth media (+ FBS) and under overnight serum starved condition (-FBS) were lysed. Total cell lysates were immunoblotted with anti-PARG antibody then with anti-PCNA as loading control.

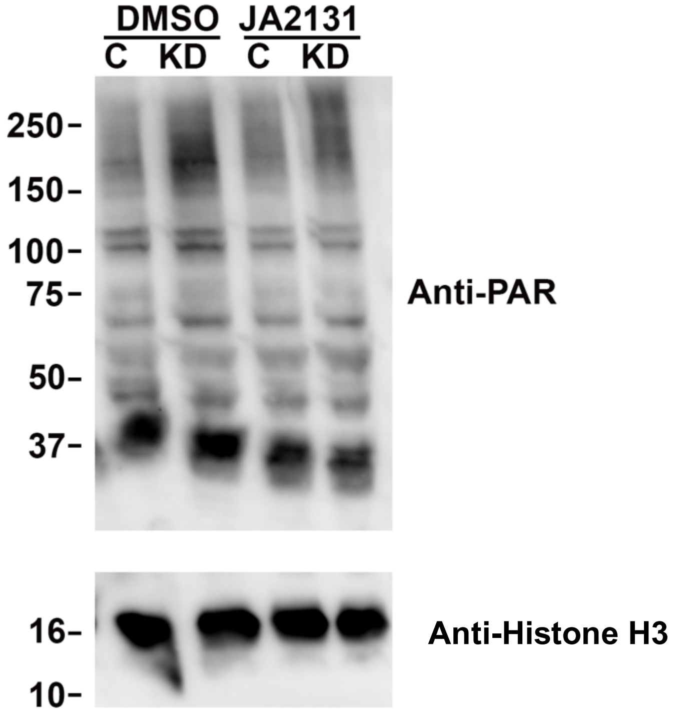

Supplementary Fig. 11: PARylation induced by PARG knockdown in MDA-MB-231 cells. Stable PARG knockdown MDA-MB-231 cells were treated with 5 μM JA2131 for two hours, followed by 7 Gy X-ray and 1 hour recovery. Total lysates were immunoblotted for anti-PAR (upper panel) followed by anti-histone H3 (lower middle). C, Stable MDA-MB-231 cells with scramble shRNA; KD, PARG knockdown stable MDA-MB-231 cells with shPARG.

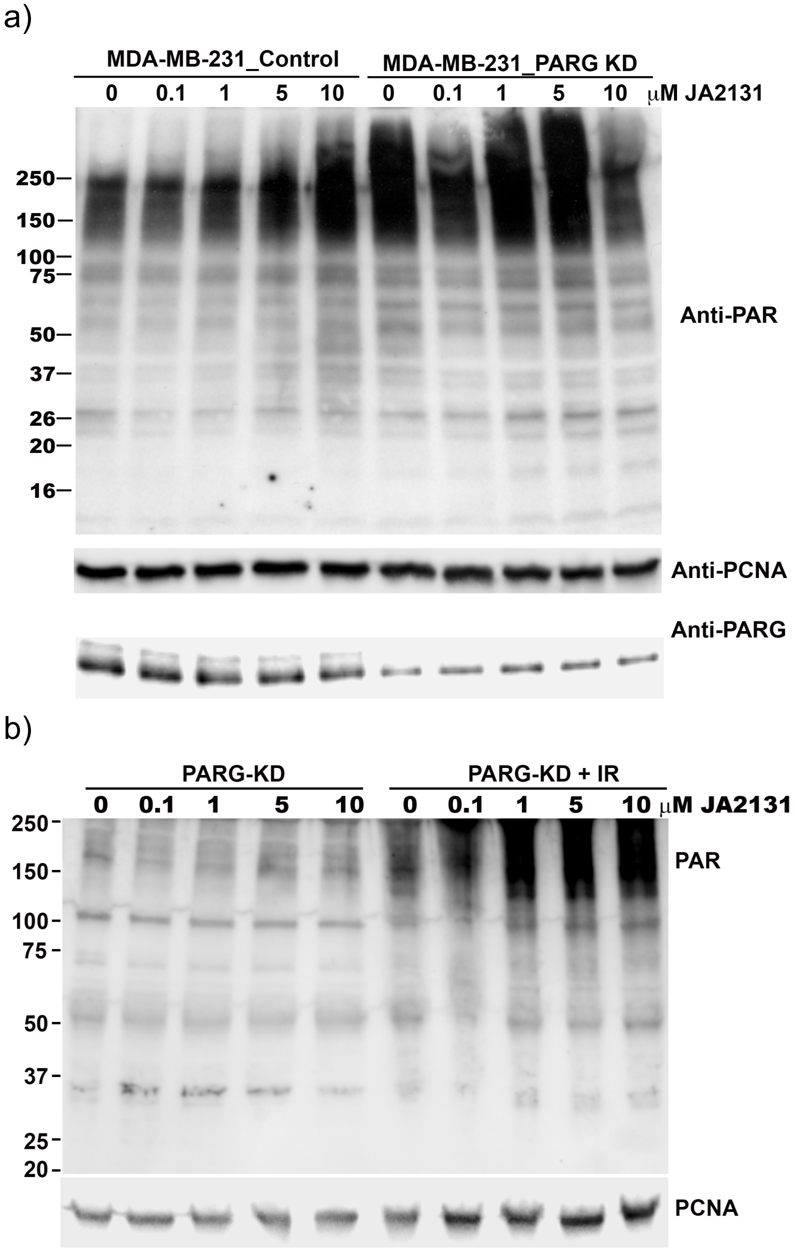

Supplementary Fig. 12: PARG knockdown cells show reduced JA2131 PAR response. (a) JA2131 dose response comparing control cells with PARG-KD. Cells were treated with indicated concentration of JA2131 for 2 hours before lysis and western blotting analysis. Anti-PCNA antibody was used as loading control and anti-PARG for expression level. (b) The same experiment as above except cells were treated with 7 Gy IR and recovered for an hour before lysis and western analysis with designated antibody.

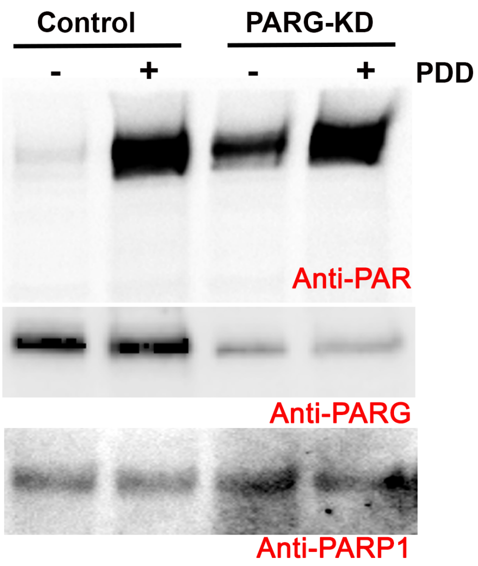

Supplementary Fig. 13: PDD induces identical PARylation regardless of PARG protein level. MDA-MB-231 (control) and MDA-MB-231 stable PARG knockdown cells (PARG-KD) were treated with 5 μM PDD (+) or left untreated (-) for two hours, followed by 7 Gy X-ray and 1 hour recovery. Total lysates were immunoblotted for anti-PAR (upper panel) followed by anti-PARG (middle middle) and PARP-1 as loading control anti-PARG (lower middle).

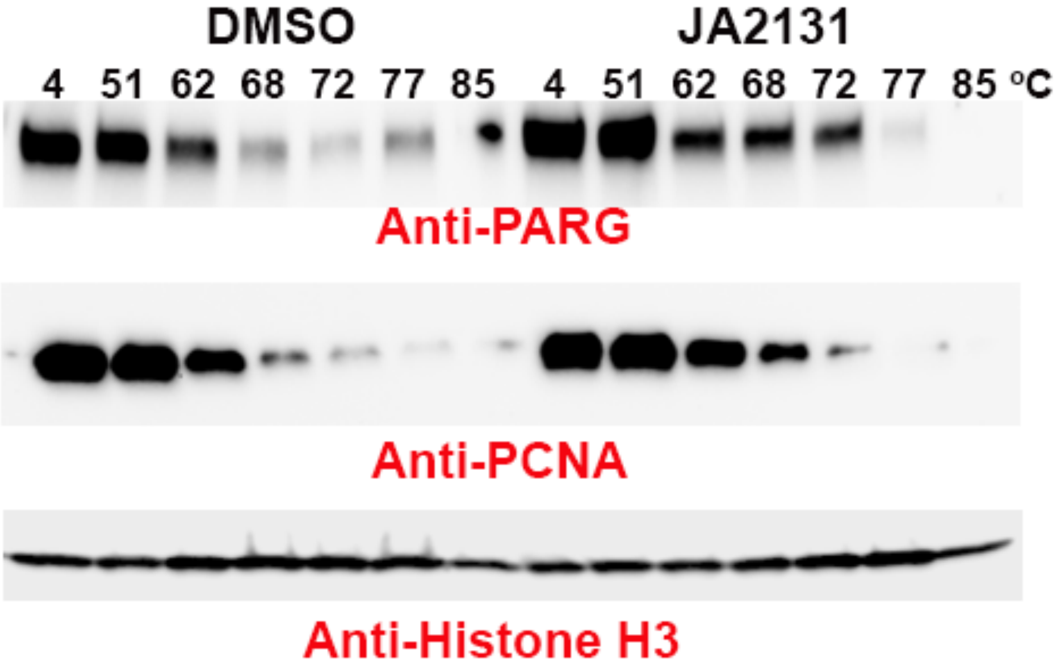

Supplementary Fig. 14: Representative CETSA western blots for PARG and PCNA. PC3 cells treated with 10 μM JA2131 or DMSO were temperature shocked as described in Materials and Methods and the resulting non-aggregated proteins were analyzed with Anti-PARG or Anti-PCNA antibody. Anti-Histone H3 was used as loading control.

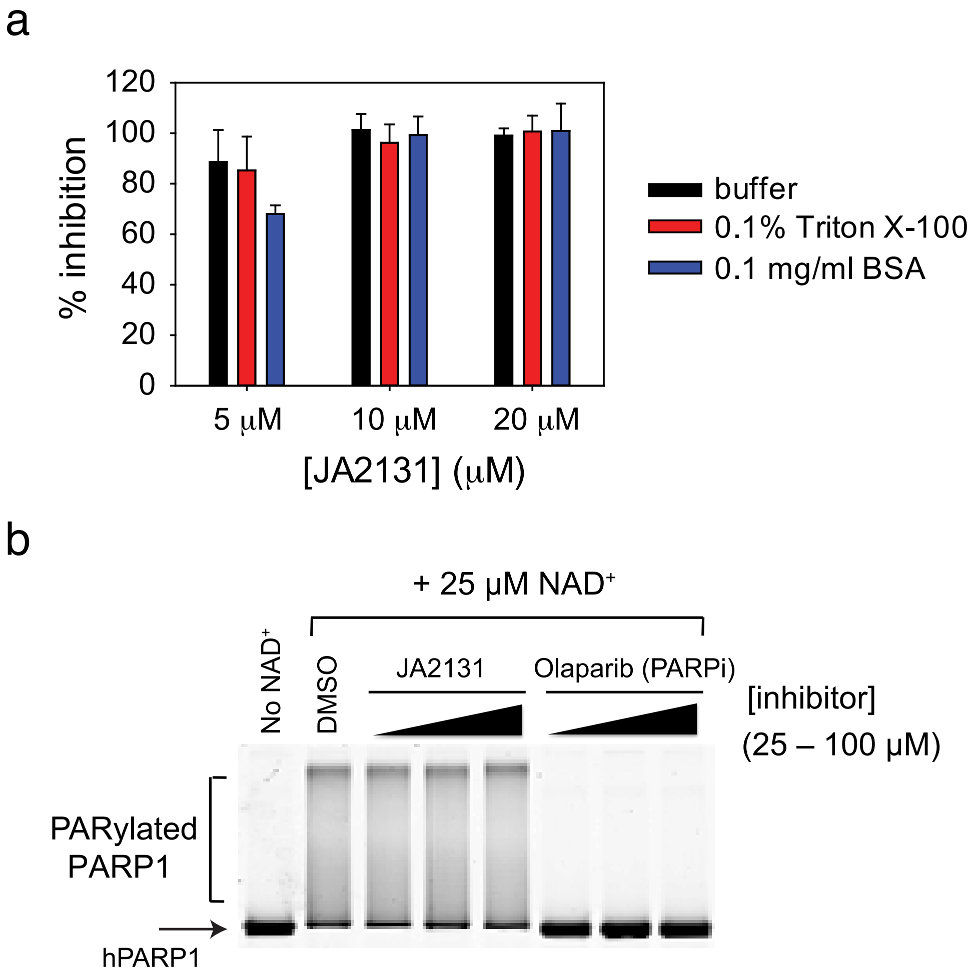

Supplementary Fig. 15: Testing JA2131 specificity. (a) *In vitro* specificity against hPARG in the presence and absence of detergent (Triton-X 100, 0.1%) and a non-specific target protein (bovine serum albumin BSA, 0.1 mg/ml). The Triton-X 100 concentration is ~10 fold higher than its critical micelle concentration (CMC, 0.0155 %) so effectively distinguished promiscuous and aggregating compounds. Neither the addition of Triton-X 100 nor BSA altered the PARG inhibition by JA2131 significantly. (b) JA2131 does not inhibit PARP1. Here, in vitro PARP1 auto PARylation was measured in the presence of increasing concentration of either JA2131 or Olaparib. The results show while Olaparib inhibits PARP1 auto-PARylation, JA2131 has no effect. This shows JA2131 has no effect in PARP1 and therefore is not an inhibitor of PARylation.

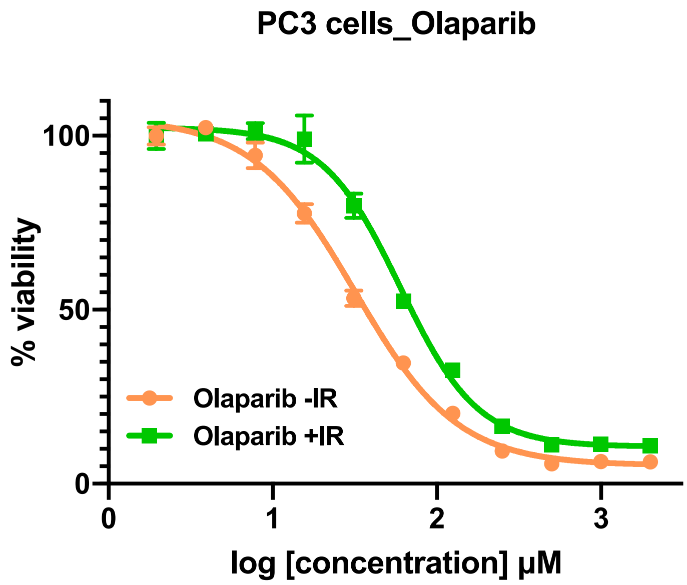

	IC_{50} (µM)
Olaparib (-IR)	31.8±4.6
Olaparib (+IR)	59.8±7.5

Supplementary Fig. 16: Cytotoxicity of Olaparib in PC3 cells. PC3 cells were treated with increasing concentration of Olaparib for an hour and then either treated with 7.0 Gy ionizing radiation (+IR) or left untreated (-IR) for 72 h. SRB assay was performed according to the protocol described in Materials and Methods. Normalized data was fitted with 4-parameter equation in GraphPad Prism 8. The resulting IC_{50} values are shown in the table below the graph. Error bars show the percent coefficient of variation.

Supplementary Note 1

PARG inhibitors as potential therapeutic option for PARP1 inhibitor resistant cancers.

As PARP1 inhibitors are now widely used in the clinic but frequently resistance is emerging, we evaluated if PARG may be a therapeutic option for patients who become PARP1 inhibitor resistant. We therefore generated PARP1 inhibitor resistant SUM149PT triple negative breast cancer cell-lines through with 100 nM talazoparib for 5 constitutive days and then with 15-50 nM talazoparib until resistant cells grew into clones. The resulting resistant cell clones were propagated and expanded to generate PARP inhibitor resistant SUM149PT derivative cells that no longer responded to PARP1i treatment. We then investigated whether the PARP resistant breast cancer cells would response to the PARG inhibitors. Four PARPi resistant SUM149PT cell clones together with wild type cells were treated with JA2131. PAR accumulation and γH2AX foci formation were analyzed by high content imaging (Fig. S6). Treatment of cells with JA2131 showed increased PAR accumulation as well as γH2AX staining seen by increased immunofluorescence signals. The PAR accumulation and γH2AX foci formation was very similar to those observed for PC3 cells (Fig. 6a) suggesting a potential new therapeutic opportunity for PARP1 resistant patients.

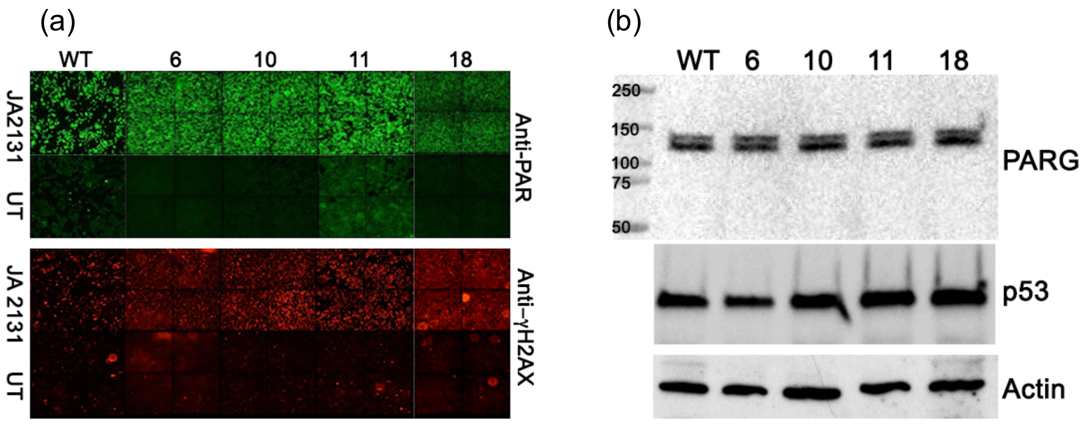

Supplementary Fig. 17: PARPi resistant cells respond to JA2131 PARGi. (a) PARGi sensitizes PARP1i resistant breast cancer cells. PARPi resistant SUM149PT cells clone 6, 10, 11, 18 together with the wild type (WT) were treated with 10 µM JA2131 for 2 hours, irradiated 3Gy ionizing radiation and then fixed, and immunofluorescence labelled for PAR and γH2AX. High content imaging shows specific accumulation of PAR and DNA damage induction observed with γH2AX signal in cells treated with PARGi. (b) Immunoblotting of total cell-lysates for PARP1i-resistant SUM149PT cells shows no change in the PARG (upper panel) and p53 (middle panel) protein expression level. Anti-actin was used as loading control (bottom panel).

Supplementary Table 1. Hits selected from the high-throughput screen of PARG inhibitors.

Hit number	NSC number	Chemotype	Selected hits showing ~ 100% PARG inhibition at both PARG concentrations (12.5 nM and 25 nM)
1	19630 (JA1-1)	JA1	✓
2	69575 (JA1-5)	JA1	✓
3	29192 (JA2-3)	JA2	✓
4	99657 (JA2-4)	JA2	
5	99667 (JA2-5)	JA2	✓
6	107684 (JA2-6)	JA2	✓
7	659162 (JA2-7)	JA2	✓
8	97999	JA2	
9	62586	JA2	
10	45741	JA2	
11	371777	JA2	
12	35334	JA2	
13	49789	JA2	
14	85433	JA2	
15	90737	JA2	
16	2805 (JA3-1)	JA3	✓
17	22907 (JA3-2)	JA3	✓
18	121838 (JA3-3)	JA3	✓
19	125034 (JA3-4)	JA3	
20	668394 (JA3-7)	JA3	
21	125908	JA3	
22	112200	JA3	
23	48693	JA3	
24	125910	JA3	
25	9608	JA3	
26	1012	JA3	
27	1011	JA3	
28	157307	JA3	
29	228148 (JA4-2)	JA4	✓
30	348401	JA4	
31	130796	JA4	
32	150289	JA4	
33	147758	JA4	
34	113997	JA4	
35	73101	JA4	
36	128884	JA4	
37	22225	JA4	
38	17173 (JA5-1)	JA5	
39	27236 (JA5-2)	JA5	

40	45382 (JA5-3)	JA5	✓
41	75140 (JA5-4)	JA5	
42	77393 (JA5-5)	JA5	
43	134196 (JA5-6)	JA5	
44	134149	JA5	
45	34238	JA5	
46	37031	JA5	
47	7223	JA5	
48	83217	JA5	
49	88947	JA5	✓
50	16168	JA5	
51	128437	JA5	

Supplementary Table 2. SAR for compounds from five representative chemotypes identified by HTS.

Name of compound	Chemical structure	Dose-response inhibition of PARG activity	Estimated IC_{50} (μM)
		JA1	
JA1-1 (NSC19630)	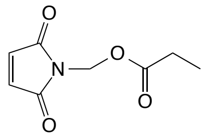	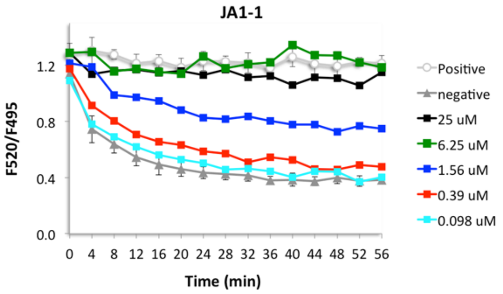	1 ~ 2
JA1-2 (NSC58206)	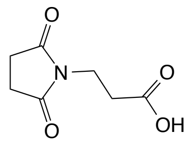	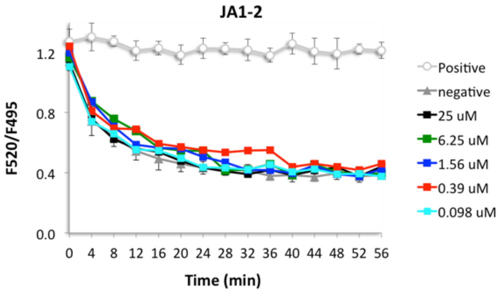	ND
JA1-3 (NSC69575)	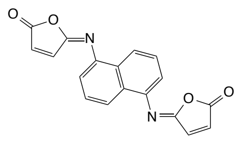	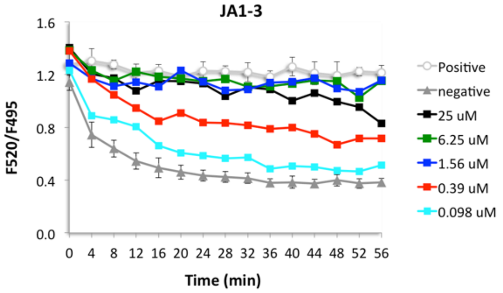	~ 0.4
JA1-4 (NSC192708)	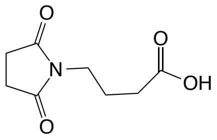	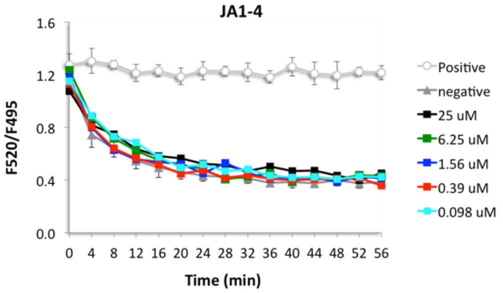	ND

JA1-5<br>(JS-2088)	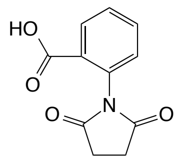	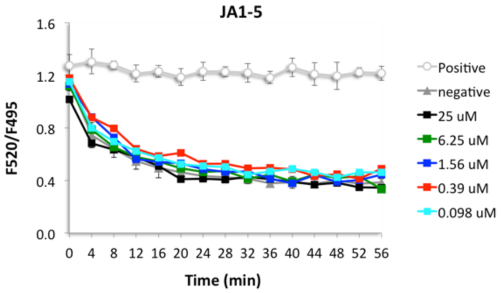	ND
		JA2	
JA2-1<br>(NSC14372)	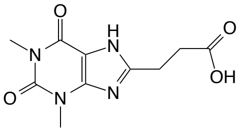	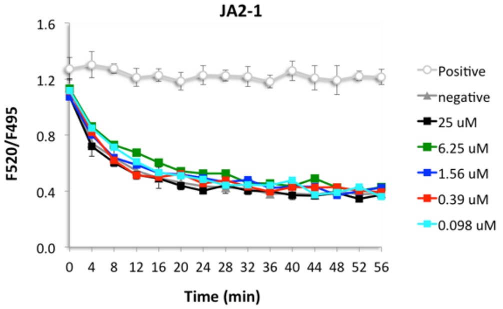	ND
JA2-2<br>(NSC14374)	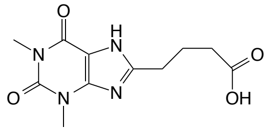	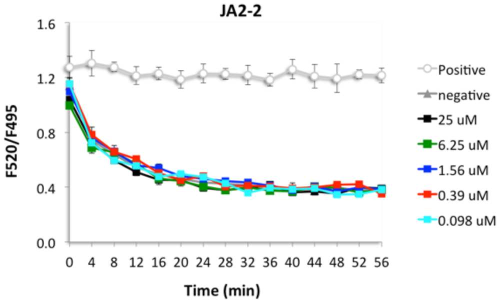	ND
JA2-3<br>(NSC29192)	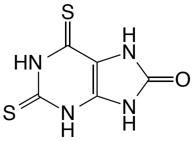	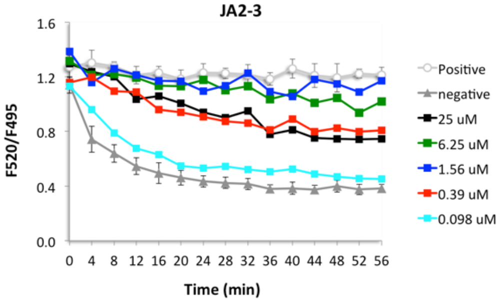	~ 0.4
JA2-4<br>(NSC99657)	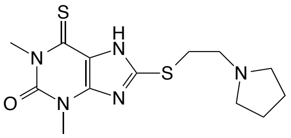	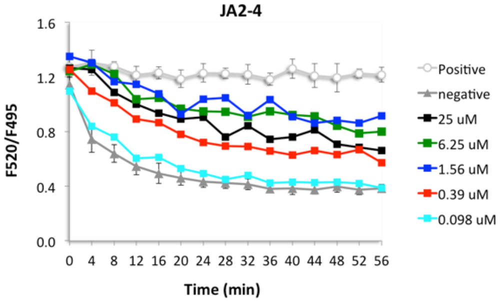	~ 0.4

<table>
<tr><td>JA2-5
(NSC99667)</td><td>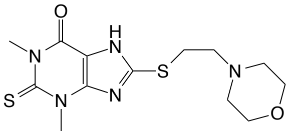</td><td>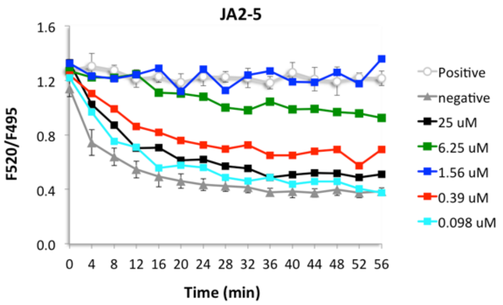
</td><td>~ 0.4</td></tr>
<tr><td>JA2-6
(NSC107684)</td><td>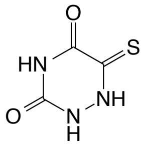</td><td>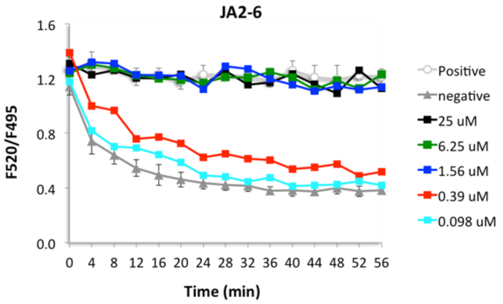
</td><td>0.5 ~ 1</td></tr>
<tr><td>JA2-7
(NSC659162)</td><td>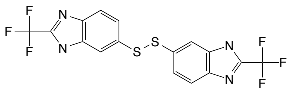</td><td>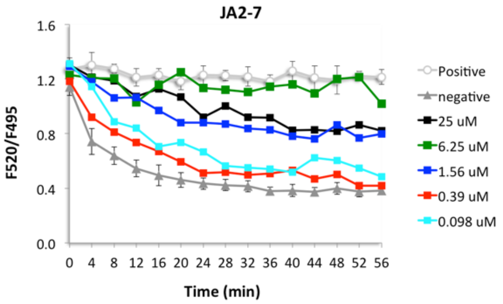
</td><td>~ 1.5</td></tr>
<tr><td>JA2-8
(JFD03560SC)</td><td>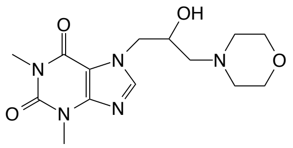</td><td>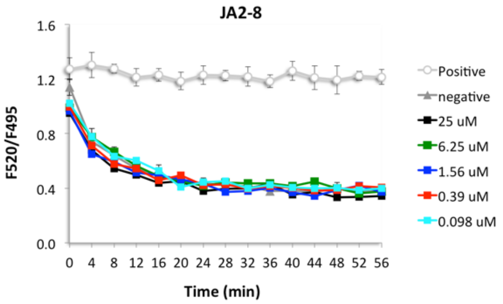
</td><td>ND</td></tr>
<tr><td>JA2-9
(Z57032584)</td><td>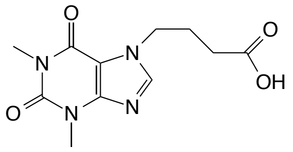</td><td>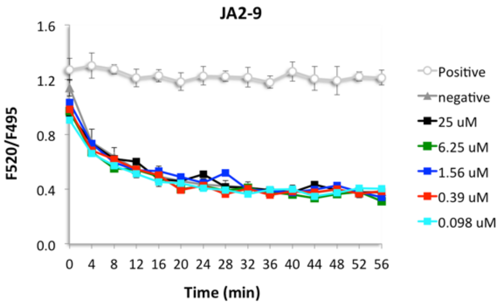
</td><td>ND</td></tr>
<tr><td colspan="4">JA3</td></tr>
</table>

<table>
<tr><td>JA3-1
(NSC2805)</td><td>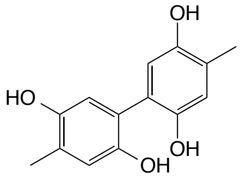
</td><td>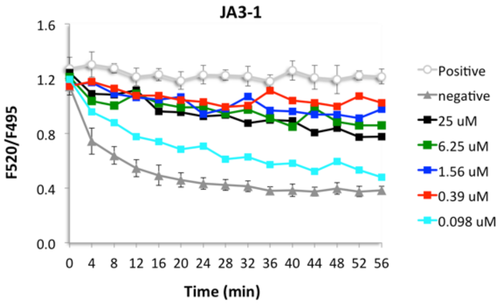
</td><td>~ 0.1</td></tr>
<tr><td>JA3-2
(NSC22907)</td><td>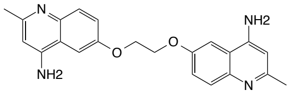
</td><td>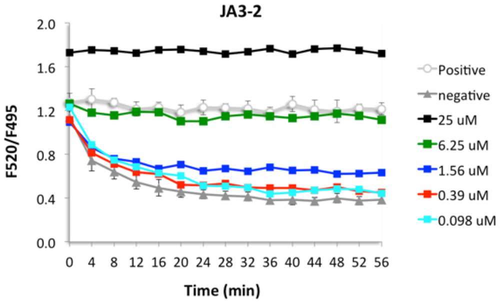
</td><td>ND</td></tr>
<tr><td>JA3-3
(NSC121838)</td><td>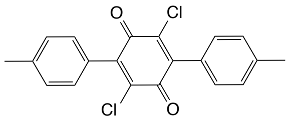
</td><td>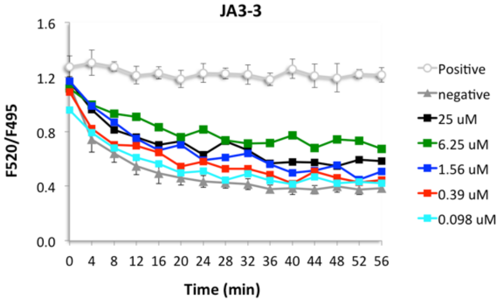
</td><td>Weak</td></tr>
<tr><td>JA3-4
(NSC125034)</td><td>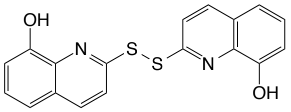
</td><td>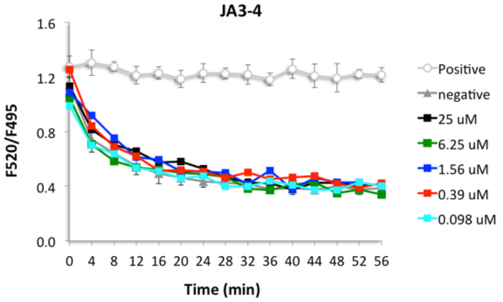
</td><td>ND</td></tr>
<tr><td>JA3-5
(NSC407988)</td><td>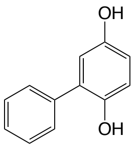
</td><td>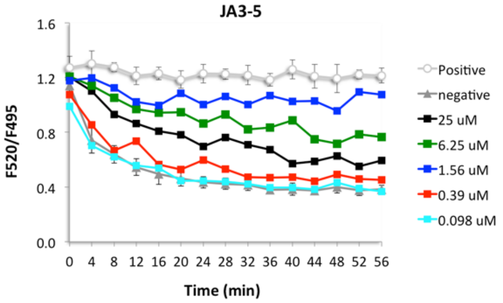
</td><td>0.5 ~ 1</td></tr>
</table>

JA3-6<br>(NSC663284)	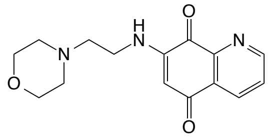	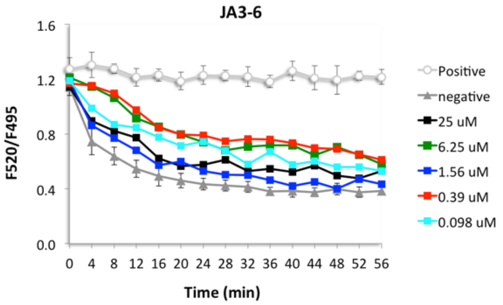	Weak
JA3-7<br>(NSC668394)	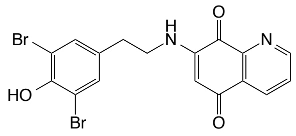	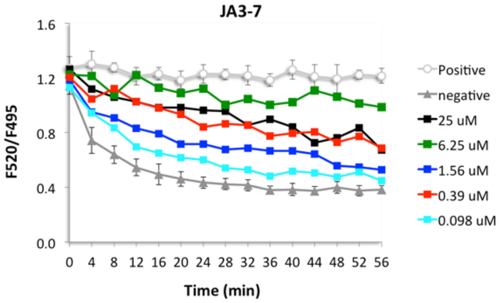	1.5 ~ 3
JA4			
JA4-1<br>(NSC228147)	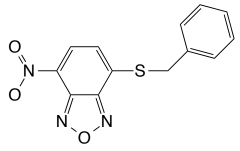	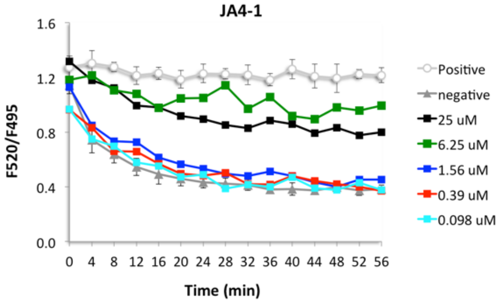	3 ~ 4
JA4-2<br>(NSC228148)	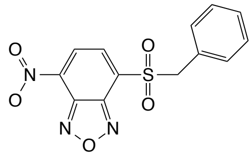	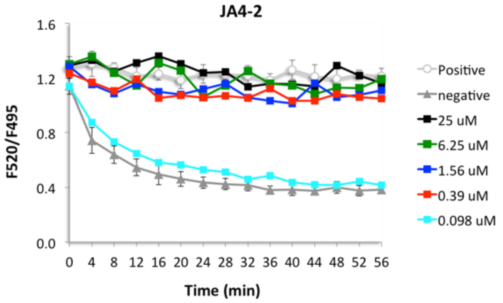	0.2 ~ 0.3
JA5			
JA5-1<br>(NSC17173)	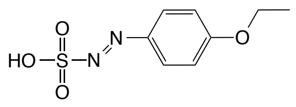	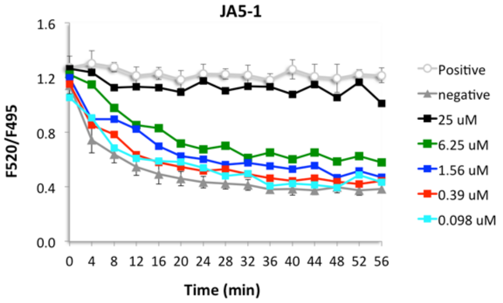	6 ~ 10

<table>
<tr><td>JA5-2
(NSC27236)</td><td>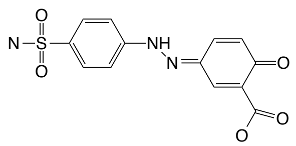</td><td>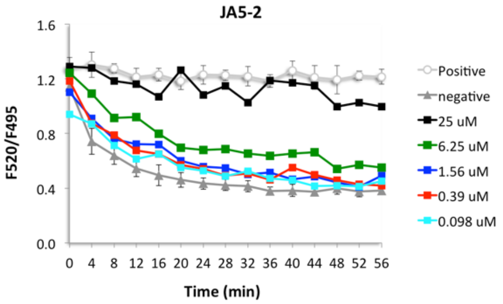
</td><td>6 ~ 10</td></tr>
<tr><td>JA5-3
(NSC45382)</td><td>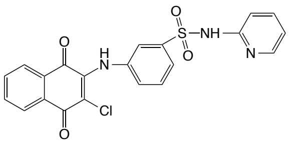</td><td>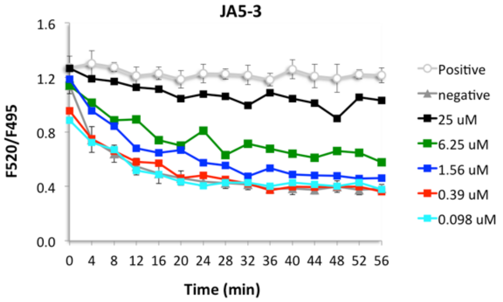
</td><td>~ 6</td></tr>
<tr><td>JA5-4
(NSC75140)</td><td>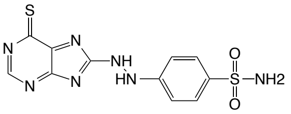</td><td>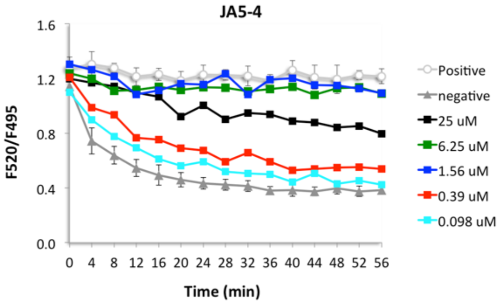
</td><td>0.5 ~ 1</td></tr>
<tr><td>JA5-5
(NSC77393)</td><td>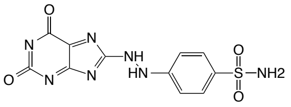</td><td>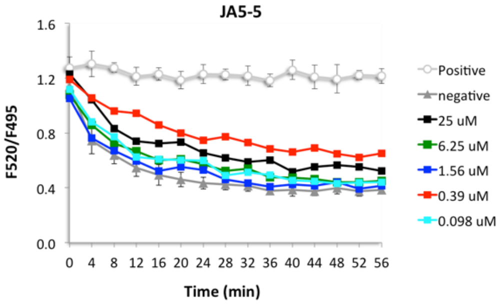
</td><td>weak</td></tr>
<tr><td>JA5-6
(NSC134196)</td><td>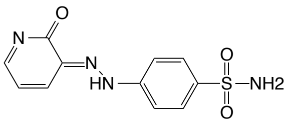</td><td>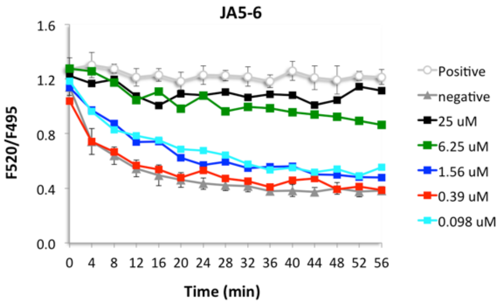
</td><td>2 ~ 4</td></tr>
</table>

JA5-7<br>(F3350-0573)	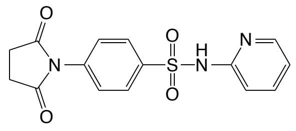	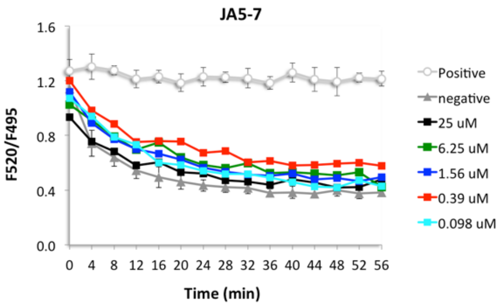	ND
JA5-8<br>(BAS 05169959)	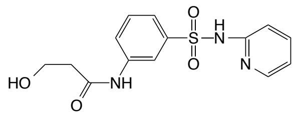	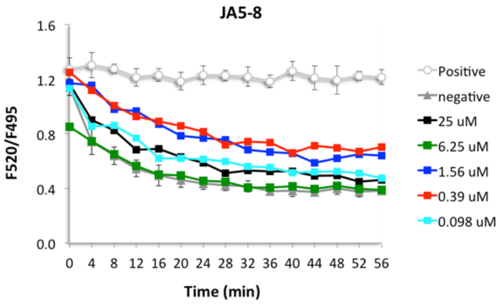	weak
JA5-9<br>(AO-476)	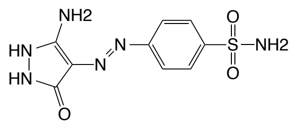	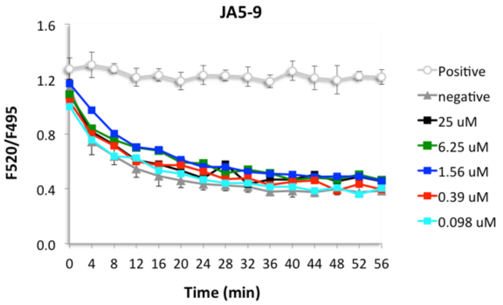	ND
JA5-10<br>(EN300-63858)	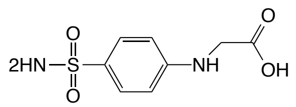	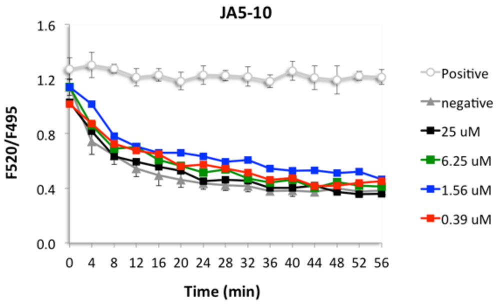	ND
JA5-11<br>(Z385453050)	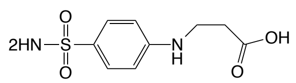	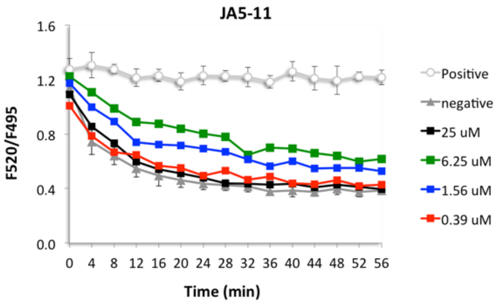	weak

Compound clusters were filtered for potentially promiscuous and reactive compounds (J.Med. Chem. 2012, 9763-9772., Aust. J. Chem. 2013, 66, 1483-1494., ACS Chem. Biol. 2018, 36-44.),

which resulted in the selection of the xanthine chemical series, JA2, The JA1 cluster is comprised of succinimides and closely related analogues. Several members of this cluster contain α, β-unsaturation (maleimide) and the liability of potential chemical reactivity. Hence this series was deprioritized. The next cluster, JA3 is comprised of predominately hydroquinone and quinone templates. The oxidizability, potential promiscuity, and fluorescence (interference with assays) associated with polyphenolic compounds renders the hydroquinone series less desirable for further investigation. Similarly, the potential chemical reactivity associated with the electrophilic nature of nitrobenzofuranzans rendered the JA4 series undesirable. The benzyl-sulfonamides series, JA5, is a compound cluster of containing hydrazines, hydrazones, and metal-chelating functional groups. We judged the risk of the chemical promiscuity and toxicity of this series too high.

Supplementary Table 3. Crystallographic data statistics of PARG/Inhibitor Complexes.

	hPARG-JA2-4 complex	hPARG-JA2131 complex	hPARG-JA2120 complex	hPARG-JA2-8 complex	hPARG-JA2-9 complex
Data collection					
Space group	$P2_1$	$P2_1$	$P2_1$	$P2_1$	$P2_1$
Cell dimensions					
a, *b*, *c* (Å)	44.6, 65.8, 88.4	44.7, 66.0, 88.6	44.6, 65.7, 88.4	44.7, 66.0, 88.6	44.7, 66.2, 88.9
α, β, γ (°)	90.0, 95.2, 90.0	90.0, 95.5, 90.0	90.0, 95.0, 90.0	90.0, 95.2, 90.0	90.0, 95.6, 90.0
Wavelength	1.54	1.54	1.54	1.54	1.54
Resolution (Å)	30 – 1.7	30 – 1.9	30 – 1.8	30 – 2.0	30 – 2.0
R_{sym} (%)	6.0 (39.8)	12.7 (46.3)	7.4 (29.7)	11.2 (58.0)	8.1 (40.1)
I / σ_I	27.0 (2.8)	11.8 (1.9)	21.7 (3.9)	12.5 (1.4)	17.5 (2.1)
Completeness (%)	97.2 (89.4)	97.3 (82.1)	93.8 (85.3)	98.4 (84.4)	99.1 (89.2)
Redundancy	5.7 (3.5)	3.8 (2.2)	4.9 (3.9)	3.9 (2.2)	4.0 (2.6)
Refinement					
Resolution (Å)	30 – 1.7	30 – 1.9	30 – 1.8	30 – 2.0	30 – 2.0
No. reflections	51558	37412	42142	32438	32975
R_{work} / R_{free}	15.5/19.5	17.9/22.5	16.8/20.3	18.4/24.1	17.1/22.1
No. atoms					
Protein	4025	4010	4008	4003	4008
Ligand/ion	21	22	22	23	19
Water	465	248	300	228	242
B-factors					
Protein	26.3	26.3	21.6	30.0	30.3
Ligand/ion	35.2	44.5	18.8	53.4	39.3
Water	39.1	30.9	28.6	34.6	34.5
R.m.s deviations					
Bond lengths (Å)	0.019	0.017	0.018	0.016	0.017
Bond angles (°)	1.94	1.80	1.82	1.72	1.69

*Values in parentheses are for highest-resolution shell.
Each dataset was collected from a single crystal.

Supplementary Table 4. Synchrotron crystallographic statistics for PARG-inhibitor complexes.

	hPARG-JA2131	hPARG-JA2120
Data Collection		
Wavelength	0.98	0.98
Resolution range	35.3 - 1.7 (1.76 - 1.7)	34.4 - 1.6 (1.66 - 1.6)
Space group	$P2_1\ 2_1\ 2_1$	$P2_1\ 2_1\ 2_1$
Unit cell		
a, *b*, *c* (Å)	66.5 89.3 94.2	66.3 89.1 94.3
α, β, γ (°)	90 90 90	90 90 90
Total reflections	1190409 (79669)	1469470 (148377)
Unique reflections	62296 (6098)	74163 (7334)
Multiplicity	19.1 (13.1)	19.8 (20.2)
Completeness (%)	1.00 (0.99)	1.00 (1.00)
Mean I/sigma(I)	17.7 (0.93)	18.6 (0.99)
Wilson B-factor	24.5	24.8
R-merge	0.139 (2.688)	0.118 (3.582)
R-meas	0.143 (2.798)	0.1211 (3.672)
CC1/2	0.999 (0.431)	1 (0.431)
CC*	1 (0.776)	1 (0.776)
Refinement		
Reflections used in refinement	62266 (6079)	74153 (7329)
Reflections used for R-free	3235 (287)	3687 (385)
R_{work}	0.1826 (0.3118)	0.1825 (0.3011)
R_{free}	0.2089 (0.3519)	0.2117 (0.3546)
Number of non-hydrogen atoms	4324	4411
macromolecules	3942	4030
ligands	22	22
Protein residues	498	499
RMS bonds (Å)	0.005	0.010
RMS angles (°)	0.81	1.02
Ramachandran favored (%)	98	97
Ramachandran allowed (%)	2.2	2.8
Ramachandran outliers (%)	0	0
Rotamer outliers (%)	0	0
Clashscore	1.03	0.13
Average B-factor	29.0	30.9
macromolecules	28.1	30.2
ligands	54.3	28.0
solvent	36.7	39.1

Statistics for the highest-resolution shell are shown in parentheses.

Supplementary Figure 18: Uncropped western blots

Un-cropped Western Blots a) Related to Figure 4a b) Related to Figure 4b

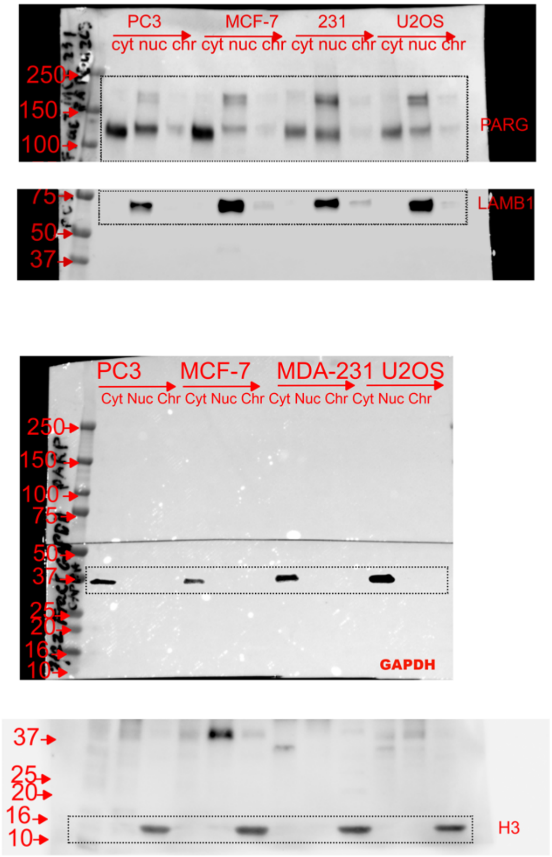

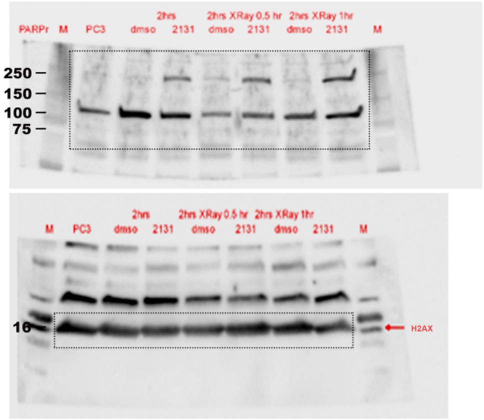

c) Related to Figure 4c

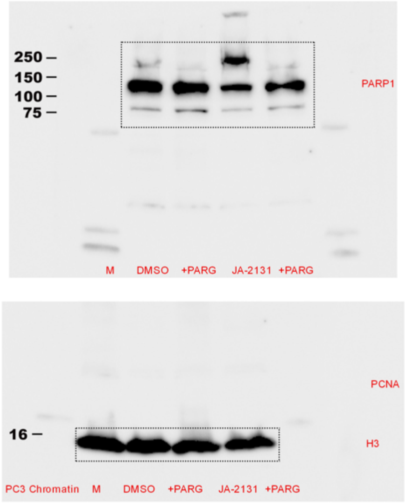

Related to Figure 6c

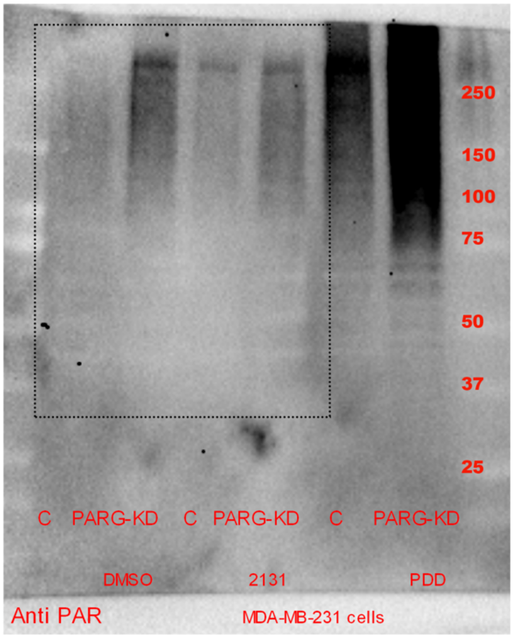

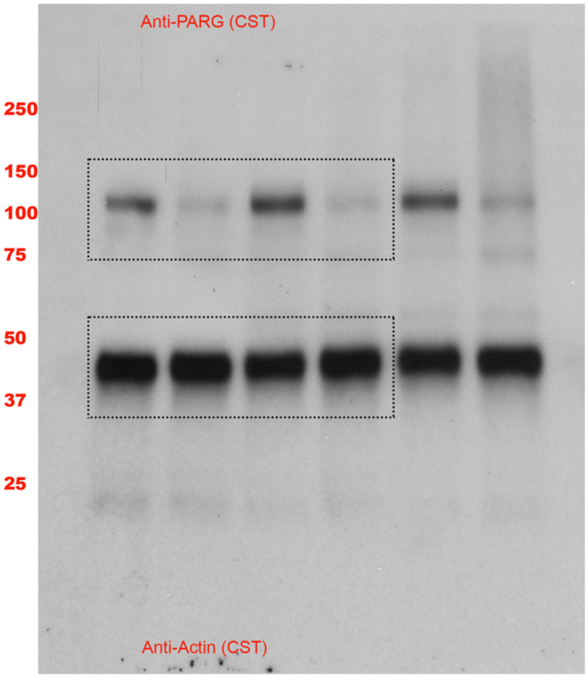

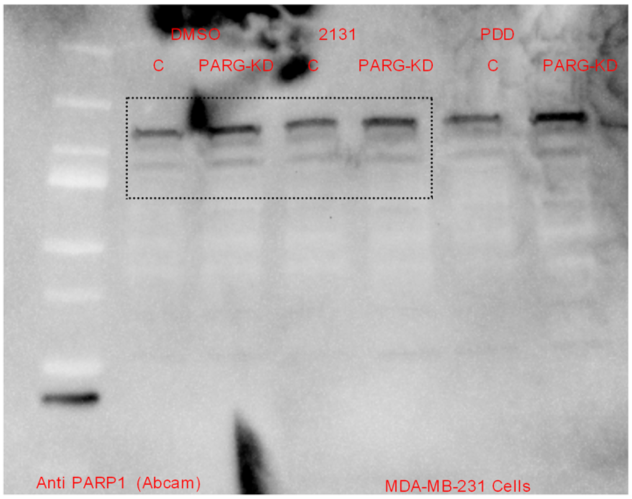

Supplement: Supplementary file 1 — Supplementary Information [file 41467_2019_13508_MOESM1_ESM.pdf]
