# Supplementary material for: Selective small molecule PARG inhibitor causes replication fork stalling and cancer cell death
Source: Nat Commun. 2019 Dec 11;10:5654. doi: 10.1038/s41467-019-13508-4 (PMC6906431; doi:10.1038/s41467-019-13508-4)
Supplement: Supplementary file 2 — Reporting Summary [file 41467_2019_13508_MOESM2_ESM.pdf]

nature research

Corresponding author(s): JOHN A. TAINER

Last updated by author(s): Sep 21, 2019

Reporting Summary

Nature Research wishes to improve the reproducibility of the work that we publish. This form provides structure for consistency and transparency in reporting. For further information on Nature Research policies, see Authors & Referees and the Editorial Policy Checklist.

Statistics

For all statistical analyses, confirm that the following items are present in the figure legend, table legend, main text, or Methods section.

n/a	Confirmed	
☐	☒	The exact sample size (*n*) for each experimental group/condition, given as a discrete number and unit of measurement
☐	☒	A statement on whether measurements were taken from distinct samples or whether the same sample was measured repeatedly
☐	☒	The statistical test(s) used AND whether they are one- or two-sided<br>*Only common tests should be described solely by name; describe more complex techniques in the Methods section.*
☐	☒	A description of all covariates tested
☐	☒	A description of any assumptions or corrections, such as tests of normality and adjustment for multiple comparisons
☐	☒	A full description of the statistical parameters including central tendency (e.g. means) or other basic estimates (e.g. regression coefficient) AND variation (e.g. standard deviation) or associated estimates of uncertainty (e.g. confidence intervals)
☐	☒	For null hypothesis testing, the test statistic (e.g. *F*, *t*, *r*) with confidence intervals, effect sizes, degrees of freedom and *P* value noted<br>*Give P values as exact values whenever suitable.*
☒	☐	For Bayesian analysis, information on the choice of priors and Markov chain Monte Carlo settings
☒	☐	For hierarchical and complex designs, identification of the appropriate level for tests and full reporting of outcomes
☒	☐	Estimates of effect sizes (e.g. Cohen's *d*, Pearson's *r*), indicating how they were calculated

Our web collection on statistics for biologists contains articles on many of the points above.

Software and code

Policy information about availability of computer code

Data collection	https://portal.gdc.cancer.gov
Data analysis	R package “ggpllot2” and “ggpubr”, Unpaired Two-Samples Wicoxon Test, Mann-Whitney test

For manuscripts utilizing custom algorithms or software that are central to the research but not yet described in published literature, software must be made available to editors/reviewers. We strongly encourage code deposition in a community repository (e.g. GitHub). See the Nature Research guidelines for submitting code & software for further information.

Data

Policy information about availability of data

All manuscripts must include a data availability statement. This statement should provide the following information, where applicable:

- Accession codes, unique identifiers, or web links for publicly available datasets
- A list of figures that have associated raw data
- A description of any restrictions on data availability

PDB

Field-specific reporting

Please select the one below that is the best fit for your research. If you are not sure, read the appropriate sections before making your selection.

☒ Life sciences ☐ Behavioural & social sciences ☐ Ecological, evolutionary & environmental sciences

For a reference copy of the document with all sections, see nature.com/documents/nr-reporting-summary-flat.pdf

Life sciences study design

All studies must disclose on these points even when the disclosure is negative.

Sample size	For TCGA, we have a range from lowest KICH 90 to highest BRCA 1206 samples. In clonogenic assays, we seeded 500 cells per well in 6-well plates.
Data exclusions	*Describe any data exclusions. If no data were excluded from the analyses, state so OR if data were excluded, describe the exclusions and the rationale behind them, indicating whether exclusion criteria were pre-established.*
Replication	Three and more replication of experiment were performed to ensure reproducibility. Yes, our experiments are reproducible.
Randomization	*Describe how samples/organisms/participants were allocated into experimental groups. If allocation was not random, describe how covariates were controlled OR if this is not relevant to your study, explain why.*
Blinding	*Describe whether the investigators were blinded to group allocation during data collection and/or analysis. If blinding was not possible, describe why OR explain why blinding was not relevant to your study.*

Reporting for specific materials, systems and methods

We require information from authors about some types of materials, experimental systems and methods used in many studies. Here, indicate whether each material, system or method listed is relevant to your study. If you are not sure if a list item applies to your research, read the appropriate section before selecting a response.

Materials & experimental systems

n/a	Involved in the study	
☐	☒	Antibodies
☐	☒	Eukaryotic cell lines
☒	☐	Palaeontology
☒	☐	Animals and other organisms
☒	☐	Human research participants
☐	☒	Clinical data

Methods

n/a	Involved in the study	
☒	☐	ChIP-seq
☒	☐	Flow cytometry
☒	☐	MRI-based neuroimaging

Antibodies

Antibodies used	For western blots, all antibodies were diluted 1:1000 dilution, except for PAR (1:500); PAR Trevigen (Techne) Catolog #4335-MC-100 Lot #20349 Clone 10HA, PARG Bethyl Laboratories, Inc. Catalog #A305-410 Lot #1 Polyclonal, PARP1 Abcam Catolog #75607 Lot #GR31904-1 Polyclonal, PCNA Santa Cruz Catalog #SC-56 Lot #E2418 Clone PC10, H2AX Sigma Catalog #PLA0023-100uL Lot #4,Mouse IgG Conjugated-HRP Cell Siganling Catlaog # 7076s, and Rabbit IgG Conjugated-HRP Cell Signaling Catalog #7074s. For immunustaining, primary antibodies; PAR and γH2AX Sigma Catalog #9718S Lot #13 Clone 20E3 were diluted to 1:250 dilution, and secondary antibodies AlexaFlour488 Mouse Polyclonal IgG Catalog #A-21202 Lot #2018296, ATTO 564 Rabbit Polyclonal IgG Rockland Antibodies & Assays Catalog #610-155-121S Lot #25 were diluted to 1:500.
Validation	All antibodies gave specific signal against their protein of interest. Some are more specific than other, but most was responding to their the protein theoretical weight.

Eukaryotic cell lines

Policy information about cell lines

Cell line source(s)	Characterized Cell Line Core Facility, MD Anderson; ATCC.Org, https://www.atcc.org
Authentication	MCF-7 and MDA-MB-231 cells were verified by Cell Line Core Facility, MD Anderson. HeLa, Prostate cancer (PC3) and SUM149PT cells were authenticated by ATCC org.
Mycoplasma contamination	These cell lines have been tested for mycoplasma, and they are mycoplasma negative.
Commonly misidentified lines (See ICLAC register)	*Name any commonly misidentified cell lines used in the study and provide a rationale for their use.*

Clinical data

Policy information about clinical studies

All manuscripts should comply with the ICMJE guidelines for publication of clinical research and a completed CONSORT checklist must be included with all submissions.

Clinical trial registration	*Provide the trial registration number from ClinicalTrials.gov or an equivalent agency.*
Study protocol	*Note where the full trial protocol can be accessed OR if not available, explain why.*
Data collection	*Describe the settings and locales of data collection, noting the time periods of recruitment and data collection.*
Outcomes	*Describe how you pre-defined primary and secondary outcome measures and how you assessed these measures.*
